# Tumor cell survival pathways activated by photodynamic therapy: a molecular basis for pharmacological inhibition strategies

**DOI:** 10.1007/s10555-015-9588-7

**Published:** 2015-10-29

**Authors:** Mans Broekgaarden, Ruud Weijer, Thomas M. van Gulik, Michael R. Hamblin, Michal Heger

**Affiliations:** Department of Experimental Surgery, Academic Medical Center, University of Amsterdam, Meibergdreef 9, 1105 AZ Amsterdam, The Netherlands; Wellman Center for Photomedicine, Massachusetts General Hospital, Boston, MA USA; Department of Dermatology, Harvard Medical School, Boston, MA USA; Harvard-MIT Division of Health Sciences & Technology, Cambridge, MA USA

**Keywords:** Apoptosis signaling kinase 1, Heat shock factor 1, ER stress, Antioxidant response, Inflammatory response, Proteotoxic stress

## Abstract

Photodynamic therapy (PDT) has emerged as a promising alternative to conventional cancer therapies such as surgery, chemotherapy, and radiotherapy. PDT comprises the administration of a photosensitizer, its accumulation in tumor tissue, and subsequent irradiation of the photosensitizer-loaded tumor, leading to the localized photoproduction of reactive oxygen species (ROS). The resulting oxidative damage ultimately culminates in tumor cell death, vascular shutdown, induction of an antitumor immune response, and the consequent destruction of the tumor. However, the ROS produced by PDT also triggers a stress response that, as part of a cell survival mechanism, helps cancer cells to cope with the PDT-induced oxidative stress and cell damage. These survival pathways are mediated by the transcription factors activator protein 1 (AP-1), nuclear factor E2-related factor 2 (NRF2), hypoxia-inducible factor 1 (HIF-1), nuclear factor κB (NF-κB), and those that mediate the proteotoxic stress response. The survival pathways are believed to render some types of cancer recalcitrant to PDT and alter the tumor microenvironment in favor of tumor survival. In this review, the molecular mechanisms are elucidated that occur post-PDT to mediate cancer cell survival, on the basis of which pharmacological interventions are proposed. Specifically, pharmaceutical inhibitors of the molecular regulators of each survival pathway are addressed. The ultimate aim is to facilitate the development of adjuvant intervention strategies to improve PDT efficacy in recalcitrant solid tumors.

## Introduction

The standard treatments for solid tumors include surgery, chemotherapy, and/or radiotherapy. However, these treatments are often associated with high morbidity and are often unsuccessful. Consequently, alternative modalities must be devised to treat solid tumors with equal or improved clinical outcomes but in a more patient-friendly manner. Photodynamic therapy (PDT) is an alternative treatment modality that entails the systemic or topical administration of a photosensitizing agent followed by local irradiation of the photosensitizer-loaded tumor tissue with light of the appropriate wavelength to match the photosensitizer absorption. Irradiation causes the photosensitizer to first enter a short-lived excited singlet state that can transition to a long-lived excited triplet state [1]. Triplet state photosensitizers can transfer energy to molecular oxygen to yield singlet oxygen (^1^O_2_) by electron transfer electrons to form superoxide anion (O_2_^•−^) and hydroxyl radicals (HO^•^). These reactive oxygen species (ROS) and their derivatives (such as lipid peroxides) subsequently oxidize biomolecules in the photosensitized tissue, causing cellular oxidative stress, tissue anoxia and tumor starvation due to ROS-mediated shutdown of tumor vasculature, and an antitumor immune response. Collectively these events contribute to cellular demise and removal of the tumor [2]. PDT provides important benefits compared to surgery, radiotherapy, and chemotherapy in that it is minimally invasive or even noninvasive and can be performed locally causing only minor damage to healthy tissue [3–5]. Moreover, PDT has been associated with increased life expectancy in cancer patients [6], is cost-effective [4, 7, 8], generally does not require extended therapeutic follow-ups, and can easily be repeated in case of cancer recurrence. The latter is often difficult or impossible with the conventional therapies.

**Fig. 1:**
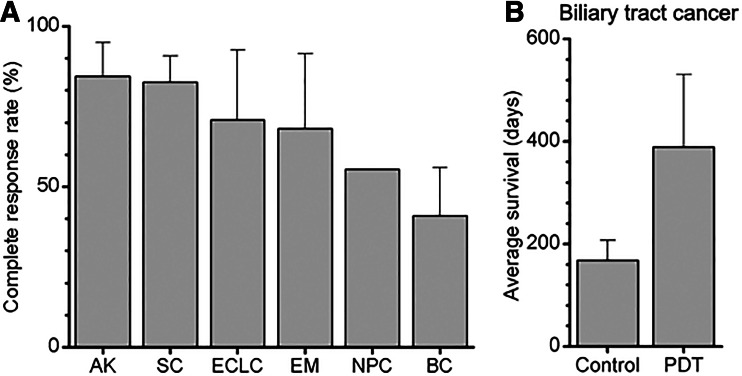
**a** Overview of clinically obtained complete response rates with PDT of actinic keratoses (*AK*), skin cancers (*SC*), early stage central lung cancers (*ECLC*), esophageal malignancies (*EM*), nasopharyngeal carcinoma (*NPC*), and bladder cancer (*BC*). SC included (nodular) basal cell carcinomas and squamous cell carcinomas [9]. EM included Barrett’s esophagus, low-grade dysplasia, high-grade dysplasia, and esophageal cancer [10]. BC included carcinoma *in situ*, recurrent superficial bladder cancer, and early stage lesions [11]. Complete response rates were averaged using the longest time interval in each study. **b** Average of the median survival time postdiagnosis of extrahepatic cholangiocarcinoma patients treated with PDT or left untreated (control) [12]. Adjuvant treatments, type of photosensitizer, light source, and light dose were not taken into account, as a result of which no statistical analyses were performed

PDT has proven to be highly effective in the treatment of various types of cancer (Fig. 1a) [9–11, 13]. However, bladder and nasopharyngeal tumors exhibit poor complete response rates following PDT (Fig. 1a) [14–16]. For a variety of esophageal lesions and early-stage central lung cancers, the results differ greatly depending on the center administering the treatment and the exact type of PDT procedure performed [10, 11]. With respect to the treatment of nonresectable extrahepatic cholangiocarcinomas, PDT has shown promising results by considerably improving the median survival of patients (Fig. 1b) [12], but the therapy is currently palliative and not curative.

The therapeutic failure in some of these cancer types likely stems from the use of photosensitizers with suboptimal optical and biochemical properties, inferior photosensitizer pharmacokinetics and/or pharmacodynamics, and variations in the tumor phenotype and genotype, which may positively influence tumor cell survival following PDT-induced oxidative damage [17]. While many investigators are looking at improving or developing new PDT strategies using chemistry or engineering approaches, relatively little research has been performed on the biology behind the therapeutic resistance, including the survival mechanisms that are triggered in cells to cope with the consequences of PDT. Several transcription factors have been identified that mediate cell survival following PDT (or approaches with similarities to PDT such as ultraviolet light irradiation). These include the members of the activating protein 1 (AP-1) transcription factor family, nuclear factor E2-related factor 2 (NRF2), hypoxia-inducible factor 1 (HIF-1), nuclear factor κB (NF-κB), heat shock factor 1 (HSF1), and transcription factors associated with the unfolded protein response (UPR).

In this review, a complete overview is provided of these pathways in terms of the activation mechanism, downstream biochemical and (patho)physiological effects, current state of knowledge regarding the involvement of these pathways in promoting tumor cell survival before and after PDT, as well as potential inhibition strategies for these pathways that can be used to increase the therapeutic efficacy of PDT.

## Photodynamic and biochemical activation of survival pathways

### ROS production through photosensitizer excitation

PDT encompasses laser or light irradiation of the tumor-localized photosensitizer at a wavelength that corresponds to the photosensitizer’s main absorption peak in the longer wavelength range of the visible spectrum (typically red light that is able to deeply penetrate tissue). Irradiation of a photosensitizer with light of a resonant frequency leads to photon absorption by the photosensitizer, resulting in the transition of an electron from the ground state (S_0_) to an energetically higher but unstable first excited state (S_1_) [18]. In most molecules, the S_1_ electron rapidly (typically in the order of a few nanoseconds) undergoes vibrational relaxation and, in some instances, molecular relaxation during its decay to S_0_ [18], producing heat and emission of a photon (fluorescence), respectively. However, S_1_ electrons in photosensitizers generally exhibit a strong tendency to undergo intersystem crossing, in which the energy of the photon is redistributed over two unpaired electrons with the same spin orientation. From this lower energy yet longer lived triplet (T_1_) state, electrons can react with molecular oxygen (O_2_) in their decay to S_0_. Two types of photochemical reactions can proceed from the T_1_ state: type I reactions are characterized by electron transfer from the photosensitizer to O_2_, yielding O_2_^•–^ [18–20]. O_2_^•–^ has a relatively low reactivity but a long lifetime (several seconds) [21] and mainly acts as a precursor radical from which secondary and tertiary radicals are formed in biological systems [22]. Type II reactions are the result of energy transfer from the T_1_ electrons to O_2_, resulting in the production of highly reactive ^1^O_2_ [18, 23]. The strong reactivity of ^1^O_2_ toward lipids, nucleic acids, proteins, and other biochemical substrates is reflected by its short biological half-life (3 × 10^−9^ s) and the small area of effect in viable cells (2–4  ×° 10^−6^ cm^2^) [24]. Additionally, since the ground state of O_2_ is the triplet state, only a minor amount of energy (94.5 kJ mol^−1^) is required for excitation to the singlet state, equivalent to the energy of a photon with a wavelength of 850 nm or shorter [18].

### Mechanisms of cytotoxicity

#### PDT-induced oxidative stress

The production of ROS occurs during irradiation of the photosensitizer. Although these primary ROS are short-lived, there is ample evidence that PDT induces prolonged oxidative stress in PDT-treated cells [25, 26]. The post-PDT oxidative stress stems from (per)oxidized reaction products such as lipids [26] and proteins [27] that have a longer lifetime and, in addition to acutely generated ROS, depletion of intracellular antioxidants [28] and, hence, further exacerbation of already perturbed intracellular redox homeostasis.

The generation of ROS and oxidative stress by PDT leads to the activation of three distinct tumoricidal mechanisms. The first mechanism is based on the direct toxicity of photoproduced ROS, which oxidizes and damages biomolecules and affects organelle and cell function. For example, 8-hydroxydeoxyguanosine is a reaction product of ROS with guanosine [29] and may contribute to the induction of DNA damage by PDT [30–38]. Furthermore, 8-oxo-7,8-dihydro-2′-guanosine is a product of RNA oxidation reactions that leads to impaired RNA-protein translation [39, 40].

With respect to phospholipids, linoleic acids are prominent targets for ROS-mediated peroxidation [41], yielding 9-, 10-, 12-, and 13-hydroperoxyoctadecadienoic acids as specific products of ^1^O_2_-mediated linoleic acid oxidation [42]. Other membrane constituents such as cholesterol, α-tocopherol, aldehydes, prostanes, and prostaglandins are susceptible to oxidation by type I and type II photochemical reaction-derived ROS [41, 43–46]. The (per)oxidative modifications of phospholipids and membrane-embedded molecules by ROS lead to changes in membrane fluidity, permeability, phase-transition properties, and membrane protein functionality [47–60]. Since many photosensitizers are lipophilic, the oxidation of membrane constituents by PDT is likely a prominent cause of cell death.

In addition to nucleic acids and lipids, most protein residues are also susceptible to oxidation by type I and type II photochemical reaction-derived ROS, which can potentially lead to rupture of the polypeptide backbone as a result of peptide bond hydrolysis, main chain scission, or the formation of protein-protein cross-links [61]. Specific amino acids such as histidine, tryptophan, tyrosine, cysteine, and methionine that may be involved in the active sites of enzymes can be oxidized. Proteins that are most abundantly modified by PDT-generated ROS include proteins involved in energy metabolism (*e.g*., α-enolase, glyceraldehyde-3-phosphate dehydrogenase), chaperone proteins (*e.g*., heat shock proteins (HSP)70 and 90), and cytoskeletal proteins (*e.g*., cytoplasmic actin 1 and filamin A α) [62]. Besides detrimental effects on protein function, oxidative modification of these biologically essential substrates disrupts the normophysiological redox state of cells, leading to oxidative stress and, in case of excessive damage or stress, cell death *via* necrosis, apoptosis (reviewed in [63]), or necroptosis [64], depending on which intracellular substrates are most affected by ROS (reviewed in [65]).

Surviving cells may activate adaptation mechanisms in order to (1) restore the intracellular redox homeostasis (antioxidant response), (2) activate a stress response that aids in survival or stimulates apoptosis (immediate early stress response), and (3) facilitate in refolding or degradation of carbonylated proteins (proteotoxic stress response). Autophagy as a result of mitochondrial or ER stress may prevent apoptotic cell death and thereby constitutes a survival mechanism in sublethally damaged tumor cells following PDT [66].

#### PDT-induced hypoxia

The second tumoricidal mechanism of PDT involves the induction of local hypoxia in the irradiated tumor bulk. The acute induction of hypoxia is a result of O_2_ depletion in consequence to the O_2_ → ^1^O_2_ or O_2_^•–^ conversion and subsequent oxidation of biomolecules during PDT [67] and the shutdown of tumor vasculature after PDT [68]. The majority of systemic first- and second-generation photosensitizers localize primarily in endothelial cells as well as tumor cells that line the tumor vasculature after short drug-light intervals [69, 70], defined as the time between photosensitizer administration and light delivery. Endothelial photosensitization in particular is associated with vasculature-damaging effects [71–74] that translate to a favorable therapeutic outcome. Prolonged hypoxia due to the destruction of intratumoral vasculature was found to be crucial in the massive induction of cell death following PDT as a result of thrombosis, hemostasis, and cessation of oxygen and nutrient supply (reviewed in [68]). A state of hypoxia or even anoxia reduces the ability of cells to generate ATP by oxidative phosphorylation [75]. As will be reviewed here, hypoxia causes cells to resort to ATP production through anaerobic metabolism to sustain cell function and restore homeostasis and promote angiogenesis to resolve the hypoxic conditions. Cells that are incapable of sustaining ATP production anaerobically due to extensive oxidative stress undergo necrotic cell death (an ATP-independent mode of cell death), which is the strongest trigger for the third tumoricidal mechanism: the antitumor immune response.

#### PDT-induced antitumor immune response

The antitumor immune response, which is triggered by a form of sterile inflammation, constitutes an important process in the post-PDT removal of the treated malignancy. Various studies in mice have shown that activation of the immune system after PDT is necessary for complete eradication of the tumor [76, 77]. The tumor cell death that occurs directly from photochemical damage or as a result of vascular shutdown-mediated hypoxia/anoxia and hyponutrition is the key precursor event for the antitumor immune response.

The PDT-treated cancer cells die as a result of necrosis, apoptosis [78], necroptosis [64], and/or autophagy [79]. In all modes of cell death, intracellular molecules are released that, following their release, act as so-called damage-associated molecular patterns (DAMPs) [80]. The released molecules also comprise tumor-associated antigens (TAAs) that are otherwise shielded from recognition by immune cells and hence are nonimmunogenic until released [81]. Accordingly, the extracellular DAMPs and TAAs alert cells of the innate and adaptive immune system of impending cellular demise and the presence of malignant tissue, respectively, and consequently trigger a sterile immune response aimed at removing the PDT-treated tumor [82]. A major advantage of the PDT-triggered oncoimmunological pathways is that these pathways can trigger an antitumor immune response mediated by antigen-specific T-cells against distant tumor cells that were not subjected to PDT (referred to as abscopal effects) [83, 84].

## Survival pathways activated in tumor cells post-PDT

**Fig. 2:**
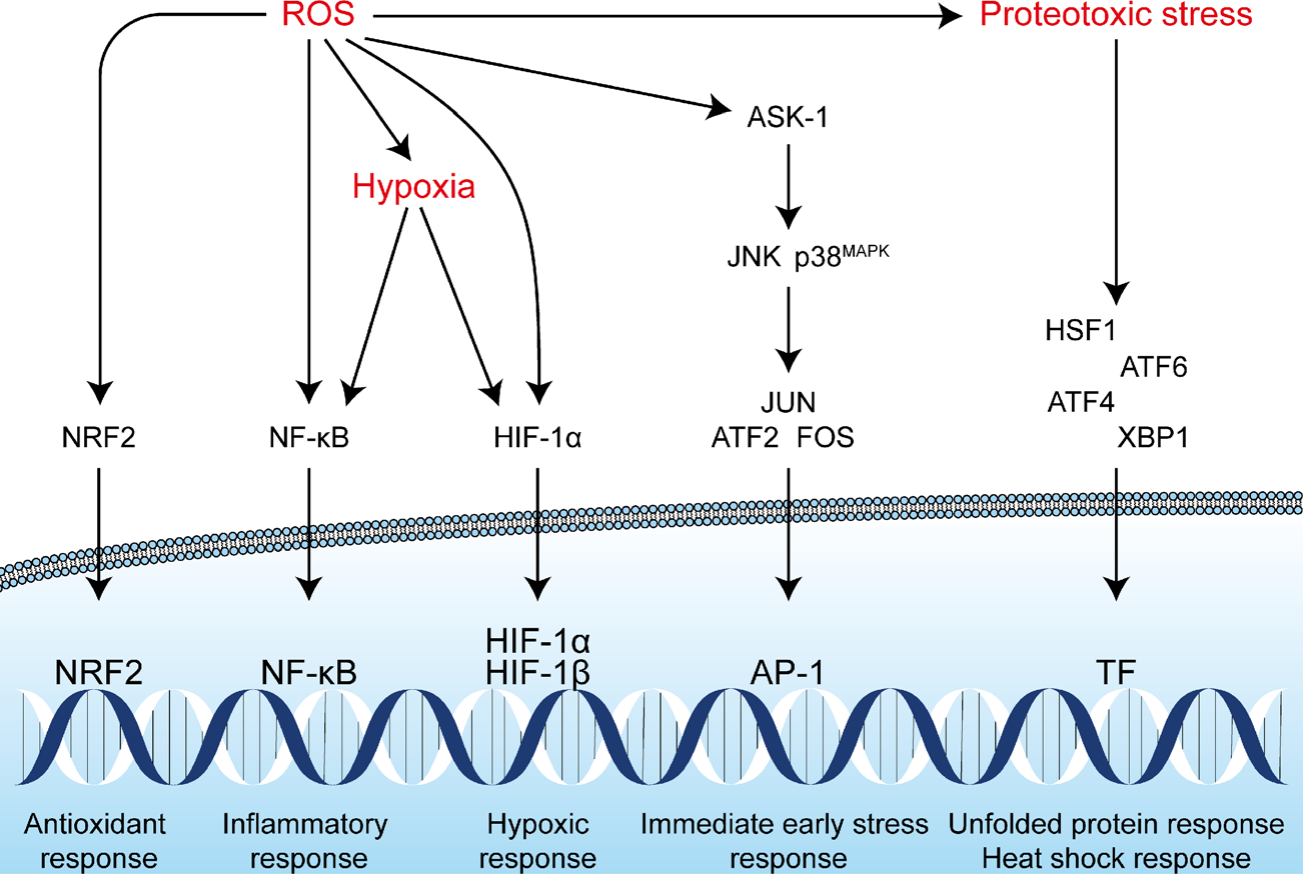
Reactive oxygen species (ROS)-induced activation of cell survival-related signal transduction pathways in cancer cells following photodynamic therapy (PDT). PDT induces vascular shutdown and oxidation of proteins, which results in hypoxia and proteotoxic stress, respectively. ROS directly trigger the NRF2-mediated antioxidant response and the ASK1-induced immediate early stress response. Hypoxia and ROS are both involved in the activation of the NF-κB inflammatory response and the HIF-1 hypoxic response. The proteotoxic stress response is characterized by the activation of several transcription factors (TF), including HSF1, ATF4, ATF6, and XBP1

The tumor cells that are subjected to sublethal oxidative damage or that are located in tumor regions not affected by vascular shutdown can activate cell survival mechanisms that have been proposed to lie at the basis of therapeutic recalcitrance [17]. We postulate that tumor cell survival following PDT is attributable to at least five interconnected pathways. These pathways include (1) an antioxidant response mediated by NRF2; (2) a hypoxic survival response mediated by HIF-1; (3) a proinflammatory and angiogenic response mediated by NF-κB; (4) a proteotoxic stress response mediated by transcription factors HSF1, X-box binding protein 1 (XBP1), activating transcription factor (ATF) 6, and ATF4; and (5) an acute stress response mediated by apoptosis signal-regulating kinase 1 (ASK1), its downstream mitogen-activated protein kinase (MAPK) that targets c-Jun N-terminal kinase (JNK) and p38^MAPK^, and the transcription factors of the activator protein 1 (AP-1) family. An overview of the survival pathways is provided in Fig. 2. The following sections will address each of these pathways individually and discuss their potential activation mechanism by PDT, their downstream effects and function, their participation in the PDT response, as well as possible inhibition strategies to reduce their cytoprotective effects and improve the tumoricidal efficacy of PDT.

Some of the survival mechanisms operate by their constitutive activation in cancer cells before PDT, which then prevent cell death following PDT. In other cases, the activation of the survival mechanisms is induced by PDT and may consequently translate to prolonged survival in cells that were subjected to sublethal oxidative damage. Despite the fact that the ROS produced by PDT are generally short-lived (Section 2.1), their secondary metabolites (*e.g*., (per)oxidized proteins, protein residues, and lipids) can sustainably disrupt cellular redox states in the tumor tissue [26, 28, 62]. This may cause a second wave of cell death, whereby the oxidatively stressed but still viable tumor cells ultimately perish *via* programmed mechanisms due to the inability to restore cell function and homeostasis [85].

The molecular pathways discussed in this chapter are generally involved in shifting the balance toward cell survival, although in some contexts, these pathways may also stimulate cell death. It should be pointed out that the exact activation mechanisms of the signaling pathways have generally not been studied in the context of PDT, but rather in the context of oxidative stress, ROS, hypoxia, or other pathways. However, since many of these activators have also been implicated in PDT, we propose that these activation mechanisms can also be applied to PDT-treated cells to explain various experimental findings that support a survival-promoting role for these pathways.

### The NRF2 pathway

During PDT, ROS are formed that oxidize a plethora of biomolecules and lead to their structural modification and dysfunction. When this occurs on an extensive scale, the oxidative stress culminates in acute cell death. However, when insufficient ROS are produced to induce acute cellular demise, cells will suffer from prolonged oxidative stress whereby the intracellular antioxidative capacity is reduced in the absence of full execution of cell death pathways. Upon exposure to sublethal oxidative stress, cells attempt to restore redox homeostasis through the upregulated production of antioxidants, detoxifying enzymes, as well as phase III drug transporters to mediate the efflux of potentially harmful oxidation products [86, 87].

NRF2 is the transcription factor that initiates this antioxidant response, a process that may be important in PDT-surviving tumor cells since it enables the cells to restore intracellular redox homeostasis in a post-PDT microenvironment and enhances the chances for long-term survival. Although NRF2 is a putative repressor of tumorigenesis by protecting cells by detoxifying ROS and ameliorating other stressors that cause malignant transformation [88], the cytoprotective effects of NRF2 are likely to contribute to reduced apoptosis- and therapy resistance in tumor cells. Moreover, NRF2 and its downstream gene products are constitutively overexpressed in many tumor types [89], especially in malignant tissues that had been exposed to the carcinogenic effects of oxygen, air pollution, and tobacco smoke [90], thereby predisposing tumor cells to tolerate PDT-induced oxidative stress to a greater extent. In a review on the role of NRF2 in oncogenesis, Gañán-Gómez *et al.* proposed that NRF2 deregulation in tumor tissue could be attributed to mutations and loss of heterogeneity; hormonal and onocogenic signaling; epigenetic, posttranscriptional, and posttranslational abnormalities; deregulation of autophagy, as well as induction by drugs [90]. Consequently, tumorigenesis is stimulated by aberrant NRF2 signaling that translates to enhanced cell growth, promotion of metastasis, increased survival, and chemoresistance [90]. Accordingly, the following sections discuss the activation mechanism of NRF2 by ROS (Section 3.1.1), the downstream gene targets of NRF2 and their function (Section 3.1.2), the evidence for the participation of the NRF2 pathway in the survival of tumor cells following PDT (Section 3.1.3), as well as potential NRF2 inhibition strategies to reduce tumor cell survival following PDT (Section 3.1.4).

#### Activation mechanism of NRF2

**Fig. 3:**
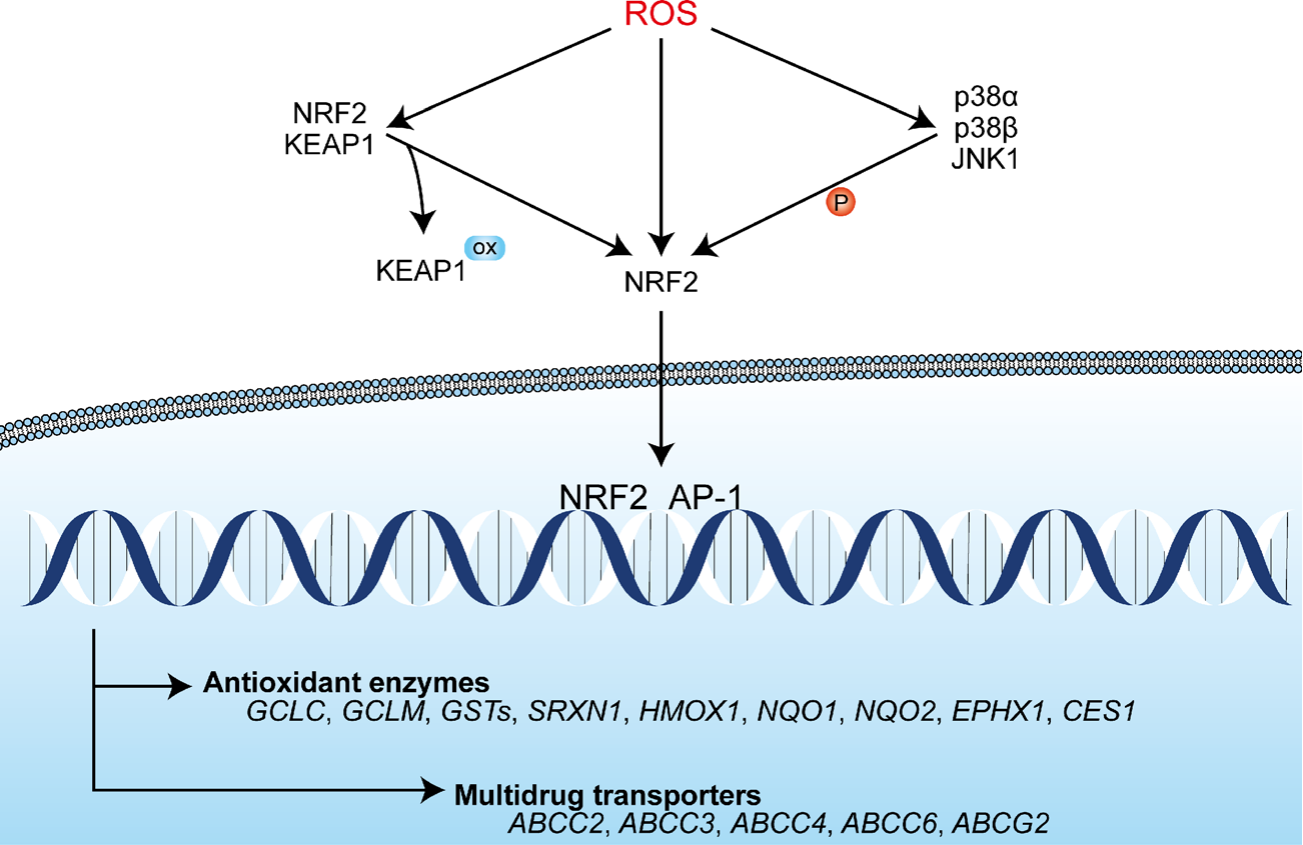
The activation mechanism of NRF2 and downstream transcription events. Under normophysiological conditions, NRF2 is sequestered in an inactive cytoplasmic complex with KEAP1. Under oxidative stress conditions, ROS mediate the oxidation (ox) of essential cysteines in the NRF2-binding domain of KEAP1, which deters complex formation. NRF2 can be additionally oxidized at Cys183 by ROS under prooxidative conditions, which enables its nuclear translocation. Moreover, ROS can activate the ASK1 pathway, in which the MAPKs JNK1 and p38α/β phosphorylate (P) NRF2 at Ser40, leading to its activation. Subsequently, NRF2 translocates to the nucleus where it dimerizes with AP-1 transcription factors (Section 3.4.2) and initiates the transcription of antioxidant enzymes (*e.g*., glutathione synthesis) and multidrug transporters (*ABCC2, ABCC3, ABCC4, ABCC6* and *ABCG2*)

NRF2 is a bZIP transcription factor that is constitutively expressed in most cells and tissue types [91–93]. Under normoxic conditions, NRF2 associates with Kelch-like ECH-associated protein 1 (KEAP1) that is bound to the cytoplasmic cytoskeleton and therefore sequesters NRF2 in the cytosol [94, 95]. Moreover, KEAP1 binds Cullin-3 that forms a scaffold for E3 ubiquitin ligases to facilitate polyubiquitination and subsequent proteasomal degradation of NRF2. Thus, under normoxic conditions, the antioxidant stress response is inactivated by high levels of cytosolic retention and degradation of NRF2 (reviewed in [86]). During oxidative stress, the NRF2-binding domain of KEAP1 is oxidized at Cys273 and Cys288, resulting in impaired KEAP1 binding to NRF2 [96]. Consequently, free NRF2 accumulates in the cytoplasm where it is activated by oxidation at Cys183, after which it is able to translocate to the nucleus [86]. Additional phosphorylation of NRF2 at serine (Ser)40 by p38α/β and/or JNK1, which are also induced by PDT (Section 3.4), may also play a role in the dissociation of the NRF2-KEAP1 complex or the prevention of NRF2-KEAP1 binding [97–99]. Once in the nucleus, NRF2 dimerizes with members of the AP-1 family, such as JUN and musculoaponeurotic fibrosarcoma oncogene homologue (MAF) subfamily proteins [100, 101], and binds to antioxidant response element (ARE) sequences to induce the transcription of antioxidant genes. An overview on the activation mechanisms of NRF2 and downstream effects is presented in Fig. 3. An elaborate review on the activation mechanisms of NRF2 is provided in [86].

#### Downstream effects of the NRF2 pathway

The products of NRF2 target genes are involved in the synthesis and redox cycling of antioxidants as well as the removal of potentially harmful oxidation products. The NRF2/AP-1 target genes include NAD(P)H:quinone oxidoreductase 1 (NQO1) and NQO2, heme oxygenase-1 (HO-1, *HMOX1*), glutamate-cysteine ligase (GCL), microsomal epoxide hydroxylase (EH-1), glutathione S-transferases (GSTs), sulfiredoxin 1 (SRXN1), and carboxylesterase 1A1 (CES1A1) [102]. EH-1 neutralizes epoxides, whereas NQO1 and NQO2 reduce oxidized quinones to prevent further cell damage by these reactive species [103, 104]. CES1A1 hydrolyzes esters and thioesters [105]. HO-1 neutralizes certain types of ROS directly as well as oxidized metabolites (lipid radicals) indirectly by producing the antioxidant molecule bilirubin from heme [106, 107]. Moreover, proteins involved in the reduction and reactivation of radical scavengers such as glutathione (GSH) and peroxiredoxins are upregulated by NRF2, including GCL (subunits GCLC and GCLM), GSTs, and SXRN1 [108, 109]. NRF2 further upregulates ATP-binding cassette subfamily C (ABCC) 2, 3, 4, and 6 (also known as multidrug resistance proteins (MRPs)) and the multidrug efflux pump ATP-binding cassette subfamily G member 2 (ABCG2), which mediate efflux of organic anions and glutathionylated, glucuronidated, and/or sulfated (bio)molecules (reviewed in [102, 110–112]). Such biomolecules are the products of oxidative stress that are potentially harmful by themselves [102]. MRPs may therefore be crucial for the detoxification of tumor cells that have survived the initial PDT-induced ROS attack and can aid in restoring the intracellular redox balance.

HO-1 in particular has been linked to cancer cell survival following PDT. Besides being upregulated by NRF2, HO-1 (encoded by the *HMOX1* gene) is upregulated by HIF-1 [113], which is also induced by PDT (Section 3.3). The function of HO-1 is to convert mitochondrially produced heme into carbon monoxide (CO) and biliverdin, of which the latter is reduced by biliverdin reductases to bilirubin [114]. Bilirubin scavenges peroxidized lipids [115, 116] and may significantly contribute to tumor cell survival following PDT by terminating lipid oxidation chain reactions. Furthermore, at low concentrations, CO possesses vasodilating, proangiogenic, anti-inflammatory, and antiapopotic properties, which can contribute to angiogenesis, tumor survival, and tumor regeneration *in vivo* [117, 118]. Although the degradation of heme to bilirubin also liberates Fe^2+^ that contributes to a prooxidant state, the release of Fe^2+^ by HO-1 was found to concomitantly increase the transcription of ferritin [119], which chelates and neutralizes free Fe^2+^ [120].

Another major pathway augmented by NRF2 is the GSH synthesis pathway, which yields an effective redox machinery aimed at scavenging ROS and neutralizing reactive intermediates such as oxidized protein residues (by glutathionylation) [121]. Synthesis of the GSH tripeptide occurs by ligation of l-glutamate and l-cysteine by GCL and addition of glycine by GSH synthetase. GSH can reduce ROS through oxidation of its thiol moiety (GSH → GS•), after which the reactive thiol is neutralized by GS-GS homodimerization (GSSG) with another GS• through disulfide bridge formation. Recycling of GSSG to GSH is catalyzed by GSSG reductase (reviewed in [121]). GSH can also react with oxidized cysteine residues, resulting in protein glutathionylation and subsequent cellular efflux *via* proteins of the MRP family [110]. Moreover, GSTs of different classes are upregulated by NRF2, which are responsible for the glutathionylation of oxidized proteins resulting in increased MRP transporter-mediated efflux of glutathionylated peptides [122]. Another role for GSTs is to inhibit molecular constituents in the ASK1 pathway, including ASK1 (by GSTM), JNK (by GSTP/GSTA), and tumor necrosis factor receptor associated factor 2 (TRAF2) (by GSTP), although the inhibitory efficacy decreases upon oxidative stress [122]. This may prevent prolonged activation of the ASK1 pathway and could stimulate cell survival as is discussed in Section 3.4. In sum, the activation of NRF2 is essential for the production of proteins involved in GSH synthesis and redox regulation, as well as the neutralization of oxidative compounds and their cellular efflux.

#### Role of the NRF2 pathway in PDT

Although NRF2 activation by ROS is well-established, its activation by PDT has been sparsely investigated. Nuclear translocation and thus activation of NRF2 was observed by Kocanova *et al*. in human bladder cancer (T24) cells and human cervical cancer (HeLa) cells following hypericin-PDT [85]. Furthermore, NRF2 target genes were overexpressed in various cancer cells after PDT, which include HO-1 [123], GCLC and GCLM subunits of GCL [124], NQO1 [124, 125], and ABCG2 [111]. The inhibition of p38^MAPK^ (p38α and p38β, Section 3.4.2) with PD169316 reduced HO-1 messenger RNA (mRNA) levels and increased the susceptibility of T24 cells to PDT [85]. These findings indicate that NRF2 is activated following PDT, that p38^MAPK^-mediated phosphorylation enhances the activity of NRF2 post-PDT, and that the expression of HO-1 by NRF2 is cytoprotective. Several reports have corroborated HO-1-mediated cytoprotection following PDT [123, 126, 127]. However, HO-1 was also found to be induced by aminolevulinic acid (ALA) prior to PDT [111], and targeted knockdown of HO-1 has been related to reduced intracellular protoporphyrin IX (PPIX) accumulation [128], indicating that HO-1 can both inhibit PS accumulation as well as reduce the PDT response. Interestingly, of the MDR proteins induced by NRF2, at least ABCG2 has been confirmed to facilitate cytoprotection against PDT by mediating the cellular efflux of photosensitizers PPIX, pyropheophorbide A, and benzoporphyrin deri-vative monoacid ring A [129] but not meso-tetrahydroxyphenylchloride and porfimer sodium [130].

#### Inhibition strategies for NRF2 and its downstream targets

Retinoic acid has been identified as an inhibitor of NRF2 in human mammary carcinoma (MCF-7) cells transfected with an ARE-luciferase reporter construct. Retinoic acid abolished the expression of genes with ARE sequences in their promoter regions [131] but did not affect the nuclear translocation or degradation of NRF2. Rather, retinoic acid inhibited the function of NRF2 by activating retinoic acid receptor α (RARα) in the nucleus. RARα sequesters NRF2 in the nucleus, thereby inhibiting the association between NRF2 and ARE sequences [131] (Table 1). Unfortunately, not much is known about the binding specificity of retinoic acid, nor has retinoic acid or any of its analogs been studied in the context of PDT. Nevertheless, retinoic acid and its analogs are also involved in the inhibition of AP-1 transcription factors (Section 3.4.2.2 Prolonged downstream effects of ASK1 activation), which constitute the main dimerization partners for NRF2. Thus, PDT with RARα activators may potentially enhance the cytotoxic effects of PDT by inhibiting both AP-1 and NRF2 survival signaling.

**Table 1:** Overview of pharmacological agents that are used for the inhibition of select targets in PDT-induced cytoprotective pathways

Pathways	Target	Inhibitor	Mechanism	Reference
NRF2	NRF2	Retinoic acid (and analogs)	Inhibition of DNA- binding due to sequestration by RARα	[131]
NRF2	HO-1	ZnPP	Nonreversible antagonist	[132]
NRF2	HO-1	SnPPIX	Nonreversible antagonist	[133]
NRF2	Cu-SOD/Zn-SOD	DDC	Chelation of Cu(II) and Zn(II)	[167]
NRF2	Mn-SOD	2-ME	Does not inhibit Mn-SOD but increases superoxide levels	[134]
NRF2	GCL	BSO	Nonreversible antagonist	[135]
NRF2	Catalase	3-AT	Nonreversible antagonist	[136]
NF-κB	RELA (NF-κB)	Parthenolide	Alkylation at Cys38, inhibition of DNA binding	[137]
NF-κB	NF-κB	Panepoxydone	Inhibition of IκB phosphorylation	[138]
NF-κB	NF-κB	Bay 11-7082	Inhibition upstream of IKK	[139]
NF-κB	NF-κB	DHMEQ	Inhibition of DNA binding and nuclear localization	[140]
NF-κB	NF-κB	α-Ketoglutarate	Reactivation of PHD proteins	[141]
NF-κB	COX-2	NSAIDs (*e.g*., ibuprofen, celecoxib)	Reversible antagonist	[142]
NF-κB	STAT3	STA-21	Inhibition of DNA binding	[143]
NF-κB	STAT3	WP1066	Dephosphorylates STAT3 and causes nuclear export	[144]
NF-κB	Survivin	LY218130B	RNAi	[145]
NF-κB	Survivin	YM155	Inhibition of transcription	[145]
NF-κB	Survivin	Terameprocol (EM1421)	Inhibition of transcription	[145]
NF-κB	IL-6/sIL-6R	sgp130Fc	Sequestration of sIL-6R	[146]
NF-κB	MMP (broad spectrum)	Prinomastat	Chelation of Zn(II) in the catalytic domain	[147]
HIF-1	HIF-1	Amphotericin B	Increased activity of FIH	[148]
HIF-1	HIF-1	Echinomycin	Inhibition of DNA binding	[149]
HIF-1	HIF-1	α-Ketoglutarate	Reactivation of PHD proteins	[141]
HIF-1	HIF-1	Curcumin	Oxidation and proteasomal degradation of HIF-1β	[150]
HIF-1	HIF-1	Acriflavine	Binding to HIF-1α dimerization domain	[151]
ASK1	AP-1	Retinoic acid (and analogs)	Inhibition of DNA binding (does not involve RARα)	[152]
ASK1	JNK1	SP600125	Reversible ATP antagonist	[153]
ASK1	p38α/β	SB202190	Reversible ATP antagonist	[154]
ASK1	p38α/β	Sb203580	Reversible ATP antagonist	[154]
ASK1	p38α/β	PD169316	Reversible ATP antagonist	[154]
UPR	HSP90	Geldanamycin (17-AAG)	ATP antagonist	[155]
UPR	HSP90	CNF2024/BIIB021	ATP antagonist	[156]
UPR	HSP90	NVP-AUY922	Complex formation with HSP70	[157]
UPR	HSP90	SNX-5422	ATP antagonist	[158]
UPR	HSP90	STA-9090	ATP antagonist	[159]
UPR	HSP70	SubA	Antagonist	[160]
UPR	HSP70	VRS-155008	ATP antagonist	[161]
UPR	HSF1	KRIBB11	Inhibition of transcriptional activity	[127]
UPR	Proteasome	Bortezomib	Antagonist of catalytic site	[162]
UPR	IRE1/ATF6	4-Phenylbutyric acid (and analogs)	Unknown	[163]
UPR	PERK	GSK-2656157	APP antagonist	[164]

In addition to inhibiting NRF2-mediated gene expression, the downstream gene products of NRF2, such as HO-1 and members of the GSH antioxidant machinery, may also be successfully inhibited by small molecular compounds (*e.g*., Zn-protoporphyrin IX (ZnPP) [132, 165], Table 1). Inasmuch as HO-1 catalyzes the degradation of heme into the antioxidants bilirubin and CO, inhibition of HO-1 with ZnPP during PDT is expected to enhance tumoricidal efficacy. Indeed, HO-1 inhibition with 2.5 μM ZnPP considerably reduced cell viability following porfimer sodium-PDT in both human (MDAH2774) and murine (C26) colon carcinoma cell lines. The addition of bilirubin or CO could not rescue cells from PDT-induced cell death upon HO-1 inhibition, suggesting a more elaborate role of HO-1 in the survival of tumor cells than merely the synthesis of antioxidants [123]. Similar results with HO-1 inhibition were obtained in WM541Lu human melanoma cells subjected to ALA-PDT, where the addition of anti-HO-1 siRNA (24 h prior to PDT) or tin-PPIX (SnPP [133]) increased the susceptibility of these cells to PDT [126]. SiRNA-mediated knockdown of HO-1 also increased the susceptibility of UM-UC-3 (but not T24, KU7, UM-CU-2, and UM-CU-4) human urothelial carcinoma cell lines to ALA-PDT [128]. With respect to other NRF2-upregulated antioxidants, Kimani *et al*. tested several inhibitors of the glutathione redox system and ROS scavenging enzymes (superoxide dismutases (SODs) and catalase) to increase the efficacy of disulfonated aluminum-phthalocyanine-PDT of MCF-7 breast cancer cells [166]. Diethyl-dithiocarbamate (DDC) and 2-methoxyestradiol (2-ME) were used as inhibitors of Cu-SOD/Zn-SOD [167] and Mn-SOD (although 2-ME has been shown not to inhibit Mn-SOD [134]), respectively (Table 1). l-Buthionine sulfoximine (BSO) was used as an inhibitor of GCL (glutathione synthesis [135]) and 3-amino-1,2,4-triazole (3-AT) as an inhibitor of catalase [136] (Table 1). All inhibitors were efficient in exacerbating PDT-induced apoptosis. The strongest effects were observed when a combination of BSO with either 3-AT or 2-ME was used, which achieved similar results as when all the inhibitors were combined. This suggests that the inhibition of H_2_O_2_ scavenging (by inhibition of catalase) and •OH scavenging (by inhibition of GCL and glutathione synthesis) and inhibition of the enzymatic dismutation of O_2_^•–^ to H_2_O_2_ (by SODs) significantly increase the susceptibility of cells to PDT [166]. In conclusion, the preemptive inhibition of both the bilirubin and glutathione synthesis pathways revealed a protective effect of these pathways on the survival of tumor cells following PDT, altogether indicating that the NRF2 pathway counteracts the cytotoxicity of PDT.

#### Concluding remarks

NRF2 is the main trigger for the antioxidant stress response that restores the intracellular redox status toward normophysiological levels in PDT-surviving cells. The antioxidant stress response is activated by oxidative stress (Section 3.1.1) and culminates in the neutralization, modification, and cellular export of oxidized/oxidizing compounds and/or potentially hazardous products of oxidation reactions (Section 3.1.2). Given the experimental evidence that NRF2 is activated following PDT (Section 3.1.3) and that inhibition of NRF2-upregulated processes potentiates the efficacy of PDT (Section 3.1.4), NRF2 seems to be an important mediator of tumor cell survival following PDT.

It is important to realize that the short-lived ROS produced during PDT cannot be scavenged by antioxidants produced downstream of the NRF2 signaling pathway since these are produced long past the half-lives of these ROS, unless there is constitutive overexpression of this pathway. Rather, NRF2 may act as an essential factor for PDT-surviving tumor cells to restore the redox imbalance and promote prolonged survival in a post-PDT microenvironment. Moreover, since NRF2-upregulated proteins HO-1, MDR1, and ABCG2 are often upregulated in many cancer types, NRF2 is likely constitutively active in tumor cells, potentially desensitizing these cells to PDT and thereby playing an instrumental role in also neutralizing the first wave of ROS directly produced by PDT. Therefore, NRF2 inhibition strategies aimed at preventing NRF2 activity prior and/or post-PDT may prove to be beneficial for the enhancement of PDT efficacy as a result of impaired tumor cell adaptation to oxidative stress.

### The NF-κB pathway

The NF-κB transcription factor family is mainly involved in the communication between tissue cells and the immune system. Both intracellular and extracellular signals are translated by NF-κB into transcriptomic responses that ultimately enable tumor cells to attract and support immune cells. NF-κB plays a role in apoptosis, inflammation, proliferation, and activation of the HIF-1 response [168]. Therefore, the activation of this pathway after PDT supports the survival of tumor cells by preventing apoptosis and promoting angiogenesis [169]. However, PDT may also repress NF-κB activity through redox modifications under severe oxidative stress as well as tumor necrosis factor α (TNF-α) signaling, which is one of the main transcriptional targets of NF-κB, that is concurrently triggered after PDT [170]. As such, NF-κB represents a complicated survival pathway that may be both activated and repressed by PDT, depending on the severity of the oxidative insult and the interaction with additional signaling pathways.

The following sections will discuss the potential activation and repression mechanisms of NF-κB (Section 3.2.1), its downstream transcriptional effects after activation, and the function of several of the upregulated proteins (Section 3.2.2). After a brief summary of the available evidence for the participation of NF-κB in the response of tumor cells to PDT (Section 3.2.3), an outline of possible inhibition strategies for NF-κB and its downstream gene products is provided (Section 3.2.4).

#### Activation mechanisms of NF-κB

**Fig. 4:**
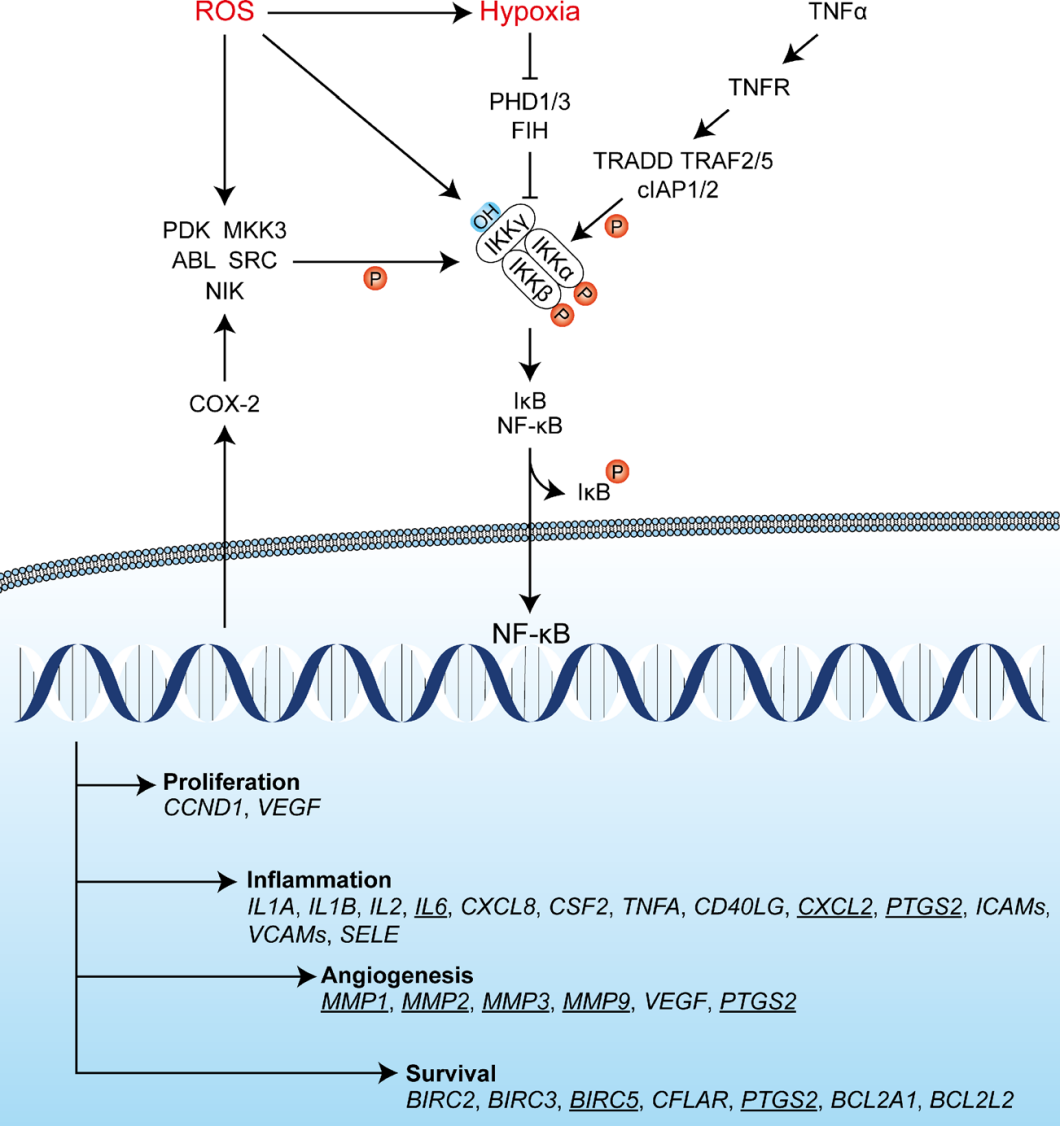
Potential activation mechanisms of NF-κB in response to PDT. ROS may activate IKK directly by oxidizing redox-sensitive cysteines on IKKγ. Alternatively, IKKα/β may be phosphorylated by kinases such as PKD, ABL, SRC, NIK, and/or MKK3 in response to oxidative stress. Hypoxia is likely a coactivator of the IKK complex, since depletion of oxygen (O_2_ → ROS) renders PHD1/3 and FIH dysfunctional, as a result of which hydroxylation of IKKγ cannot occur and IKKγ is no longer targeted for proteasomal degradation by VHL-mediated polyubiquitination. Finally, in the presence of TNF-α, the TNFR becomes activated and triggers the assembly of a complex in which TRADD, TRAF2/5, and cIAP1/2 promote the phosphorylation of IKKα/β. A stabilized and activated IKK complex phosphorylates IκB, which dissociates from the NF-κB complex and relieves its sequestration in the cytoplasm. Upon release, NF-κB translocates to the nucleus to induce a transcriptional response that promotes proliferation, inflammation, angiogenesis, and survival. *Via* COX-2, EGFR signaling activates of a variety of kinases (*e.g*., PKD, MKK3, ABL, SRC, NIK) that in turn phosphorylate and activate the IKK complex. Active NF-κB transcription factors induce the transcription of genes involved in proliferation, inflammation, angiogenesis, and survival. The *underlined genes* have been extensively investigated in relation to PDT and are discussed in detail in the main text. *CCND1* encodes cyclin D1, *PTGS2* encodes COX-2, and *BIRC5* encodes survivin

NF-κB comprises a family of proteins that include reticuloendotheliosis (REL) A, RELB, and c-Rel, as well as NF-κB1 and NF-κB2 [171, 172]. Two types of heterodimeric complexes can be formed from these proteins, each induced by different stimuli. NF-κB transcription factors composed of RELA, c-REL, and NF-κB1 are activated in the presence of proinflammatory cytokines and/or hypoxia. NF-κB complexes composed of RELB and NF-κB2 are induced solely by TNF-α. Both complexes mediate the transcription of similar target genes that contain κB elements in their promoter region and thus initiate an inflammatory response to, *e.g*., ROS and TNF-α [172]. Under normal conditions, NF-κB transcription factors are retained in the cytosol by inhibitors of κB (IκB) [168]. NF-κB is activated when IκB is phosphorylated by the IκB kinase (IKK) complex at Ser32 and Ser36, which results in the ubiquitination and degradation of IκB and corollary release and nuclear translocation of NF-κB [172]. Accordingly, the IKK complex plays a major role in the activation of NF-κB. The IKK complex is able to deactivate the IκB protein in response to three independent factors, namely in response to ROS, hypoxia, and TNF-α (Fig. 4).

##### NF-κB activation by ROS

ROS is a primary activator of NF-κB *via* oxidation of the IKK complex. IKK is composed of two subunits with kinase activity, termed IKKα and IKKβ, which are held together by one or two regulatory subunits called IKKγ (or NF-κB essential modulator, NEMO) [172]. The exact mechanism underlying IKK activation by ROS is relatively unclear and appears to be cell type-specific. In CME and Jurkat T-lymphocytes, H_2_O_2_ treatment induced phosphorylation of IκB *via* IKK, of which the activity was dependent on SH2-containing inositol 5′-phosphatase 1 (SHIP-1) [173]. However, in various human cancer cell lines and ROS-inducing treatments, the ROS-dependent phosphorylation of IKK involves protein kinase D (PKD), sarcoma (SRC), and Abelson murine leukemia viral oncogene (ABL) [174, 175], c-SRC [176], MAPK kinase (MKK)3 [177] (downstream of ASK1, Section 3.2.4), or NF-κB inducing kinase (NIK) [178]. Additionally, IKKγ contains two redox-sensitive cysteines (Cys54 and Cys347) that may be instrumental in the formation of IKKγ dimers in the presence of ROS, leading to enhanced complex formation and IKKα/β phosphorylation [179] (reviewed in [180]).

##### NF-κB activation by hypoxia

A second mechanism that may contribute to NF-κB activation following PDT is hypoxia. The hypoxic response leading to increased NF-κB activity is quite similar to the activation of HIF-1 in that the inhibition of the hydroxyl transferase activity of prolyl hydroxylase domain proteins (PHD)1 and PHD3 and possibly, but less prominently, factor inhibiting HIF-1 (FIH) play a role in the activation of IKK (reviewed in [181]). Although unequivocal experimental evidence is currently lacking, NF-κB activation by hypoxia is most likely facilitated by the hydroxylation of IKK. Similar hydroxylation-sensitive sites that were found on HIF-1α have been identified on IKKα and IKKβ [182], which interact with Von Hippel-Lindau (VHL) protein and subsequently promote polyubiquitination and proteasomal degradation [183]. Thus, it appears that PHD1 and PHD3 are able to hydroxylate and inactivate IKK complexes during normoxia and that this inactivation capacity is lost during hypoxia, potentially resulting in reduced proteasomal degradation of IKK. It should be noted that reduced degradation of the IKK complex may not be sufficient to confer NF-κB activation and that it has been proposed that hypoxia does not play a major role in the activation of NF-κB [168]. However, hypoxia may act in a costimulatory manner for the ROS- (Section 3.2.1.1 NF-κB activation by ROS) or TNF-α-mediated activation of NF-κB [181] (Section 3.2.1.3 NF-κB activation by TNF-α).

##### NF-κB activation by TNF-α

Thirdly, NF-κB can be induced *via* paracrine/autocrine signaling by TNF-α from tumor cells and chemoattracted immune cells. TNF-α is a downstream gene target of both AP-1 and NF-κB and thus provides a positive feedback loop for NF-κB activation as well as activation of the ASK1 pathway (Section 3.4). TNF-α binds the TNF receptor (TNFR) that subsequently forms activated homodimers. A signaling complex is formed by the recruitment of TNFR associated with death domain (TRADD), TRAF2/5, and cytosolic inhibitor of apoptosis (cIAP)1/2. The complex associates with a linear ubiquitin assembly complex that polyubiquitinates RIP1 and IKKγ, forming an active IKK complex. The active IKK complex is further activated by ubiquitinated RIP1, which in turn leads to the activation of TGF-β-activated kinase 1 (TAK1) and consequent phosphorylation of IKKα and IKKβ. The activated IKK complex also phosphorylates and inactivates IκB, triggering the release and nuclear translocation of the NF-κB transcription factor complex (reviewed in [184]).

##### NF-κB inhibition by ROS and TNF-α during severe oxidative stress

In contrast to the activatory capacity of ROS and TNF-α described above, severe forms of oxidative stress and/or the combination of oxidative stress and TNF-α signaling inhibit the activity of NF-κB and promote cell death. Whereas minor or moderate levels of oxidative stress lead to NF-κB activation (Section 3.2.1.1 NF-κB activation by ROS), severe oxidative stress has a detrimental effect on NF-κB activity [185]. Critical cysteines in NF-κB complexes, such as Cys62 on RELA, are susceptible to oxidation and subsequent glutathionylation or nitrosylation, which impairs DNA binding and transcriptional activity [186, 187]. Additionally, IKKα and IKKβ contain redox-sensitive Cys179, which can be oxidized by H_2_O_2_ and reduce IKK kinase activity [188]. These findings suggest that antioxidants produced *de novo viae.g*., the NRF2 pathway may facilitate NF-κB activation following a severe prooxidative insult such as PDT by ameliorating the oxidative stress, although more research is required to corroborate this claim. TNF-α exerts its anti-NF-κB effects primarily *via* mitochondrial ROS production, which may elevate the extent of preexisting moderate oxidative stress to severe oxidative stress and consequent NF-κB inhibition *via* the abovementioned processes. For example, TNF-α treatment was shown to cause oxidative stress, the cytotoxicity of which could be repressed by the addition of antioxidants [189]. Inhibition of NF-κB by TNF-α-induced oxidative stress stimulates cell death *via* prolonged activation of JNK1, given that NF-κB target gene products such as A20 and growth arrested and DNA damage (GADD)45β typically inhibit JNK1 activity. As such, ROS have been considered to act as a secondary messenger in TNF-α-induced cell death (reviewed in [185]).

The ROS-dependent activation of the NF-κB pathway has several important biological and clinical implications for PDT. Laser irradiation of tissue is characterized by light intensity attenuation with increasing depth as a result of light scattering and absorption [190], resulting in fluence gradients during PDT. Inasmuch as the extent of ROS production is proportional to the fluence [78], the cancer cells in the more distally located regions of the tumor may exhibit less ROS generation during PDT and hence are subject to a lower degree of oxidative stress than the tumor cells most proximal to the light source. Accordingly, irradiation of bulky tumors may yield a fraction of cancer cells that undergoes cell death without the activation of ROS-triggered survival pathways, whereas another fraction of cancer cells, located mainly at the deep periphery of the target tissue, may suffer from oxidative stress but survive as a result of ROS-mediated activation of *e.g*., NF-κB-mediated survival pathways. The latter fraction of cancer cells is particularly important therapeutically inasmuch as these cells may cause tumor regrowth and metastasis after PDT.

#### Downstream effects of the NF-κB pathway

The different NF-κB transcription factor complexes essentially share the same target genes that are associated with cell proliferation, inflammation, angiogenesis, and survival [172] (Fig. 4). NF-κB transcription factors induce cell proliferation (upregulation of cyclin D1 (encoded by *CCND1*) and VEGF); cause inflammatory cells to be recruited toward the tumor site (*via* the production and secretion of interleukin (IL)-1α/β, IL-2, IL-6, IL-8 (*CXCL8*), granulocyte-macrophage colony stimulating factor (GM-CSF, *CSF2*), TNF-α, cluster of differentiation 40 ligand (*CD40LG*), chemokine C-X-C motif ligand (CXCL) 2, and cyclooxygenase 2 (COX-2, encoded by prostaglandin synthase 2 (*PTGS2*)); trigger angiogenesis by upregulation of matrix metalloproteinases (*MMPs*), *VEGF*, and *PTGS2*; and facilitate inflammatory cell binding *via* selectin E (*SELE*), intercellular adhesion molecule (*ICAMs*), and vascular cell adhesion molecule (*VCAMs*) [168, 172, 191–193]. The role of NF-κB target gene products ICAM and VCAM appears to be controversial insofar as PDT reduced gene and protein expression levels despite activation of NF-κB [194, 195]. Of the inflammation-associated proteins, IL-6 plays an important role in tumor cell survival following PDT, as discussed in Section 3.2.2.4 IL-6, whereas TNF-α is also directly responsible for inducing cell death *via* apoptosis and necrosis pathways, as discussed in Section 3.2.2.3 TNF-α. To ensure survival of immune cells in a hypoxic environment, NF-κB desensitizes cells to apoptosis through the upregulation of cIAP1 (baculoviral inhibitor of apoptosis repeat-containing 2, *BIRC2*), cIAP2 (*BIRC3*), and survivin (*BIRC5*) as well as CFLAR, COX-2, and antiapoptotic members of the BCL2 family (*BCL2A1*, *BCL2L1*) [192, 196]. Especially survivin and COX-2 have been implicated in cell survival following PDT (Sections 3.2.2.1 COX-2 and 3.2.2.2 Survivin). In addition to these antiapoptotic proteins, NF-κB triggers *HIF1A* transcription that promotes immune and tumor cell survival in a hypoxic environment as a result of the upregulated production of HIF-1 transcription factor [197] (Section 3.3). NF-κB further initiates a negative feedback loop toward its own activity by inducing the expression of IκB subunits and the NF-κB inhibitor A20 [172, 198].

Overall, NF-κB stimulates tumor cell survival by inhibiting apoptosis and recruiting the immune system to facilitate angiogenesis and promote cell proliferation. The induction of NF-κB and the consequent production of cytokines may also be essential to the antitumor immune response (Section 2.2.3), which is essential for complete tumor eradication [76, 77] and long-term deterrence of tumor regrowth [199].

##### COX-2

COX-2 (encoded by *PTGS2*) is overexpressed in many types of cancer and is generally associated with reduced patient survival [200]. The promoter sequence of COX-2 contains binding sites for NF-κB, HIF-1, ATF2, FBJ murine osteosarcoma viral oncogene homologue (FOS), and JUN [201–203], making it a downstream target of three major survival pathways that are induced by PDT. The main function of COX-2 is to convert arachidonic acid to prostaglandin H_2_ (PGH_2_), which is further metabolized into PGE_2_, PGF_2α_, PGI_2_, and thromboxane A_2_ (TBA_2_) [204]. PGE_2_ induces growth of tumor epithelial cells by binding the PGE_2_ receptor and activating rat sarcoma protein (RAS) and phosphatidyl inositol 3 kinase (PI3K), which activate signaling pathways that ultimately lead to proliferation and cell division [205–207]. In addition, prostaglandins induce SRC, epidermal growth factor receptor (EGFR), MMP2, and C-C chemokine receptor 7 (CCR7) to stimulate cell migration [208–210]. Prostaglandins also stimulate angiogenesis by facilitating the production of VEGF, fibroblast growth factor (FGF)2, and molecules involved in immune cell chemotaxis and adhesion, including chemokine C-X-C motif ligand (CXCL)1, integrin αVβ3, chemokine C-C motif ligand (CCL)2, and CXC receptor 4 [207, 211–213].

##### Survivin

Survivin is a member of the inhibitor of apoptosis protein (IAP) family, which also comprises NLR family apoptosis-inhibitory protein, cIAP1, cIAP2, X-linked IAP (XIAP), and livin [214]. The expression of the genes that encode these proteins (*BIRC1*-*4* and *BIRC7*) is generally induced by transcription factor 4, signal transducer and activator of transcription 3 (STAT3), as well as the PDT-induced transcription factors NF-κB and HIF-1 (reviewed in [215]). Survivin is considered a nodule protein; a protein that stands at the center of many signaling pathways and plays a role in many cellular processes. In general, survivin stimulates cell division in the mitotic phase of the cell cycle and suppresses apoptosis (reviewed in [145]). Survivin also partakes in a chromosomal passenger complex that binds kinetochores and stimulates spindle formation to facilitate chromosome segregation during mitosis. The antiapoptotic role of survivin is reflected by its inhibition of caspase 9 [216] and prevention of XIAP degradation [145, 217]. Furthermore, alternatively spliced variants of survivin have been reported to interact with BCL2 and inhibit caspase 3 and BCL2-associated X protein (BAX) activity [218]. These proliferative and cytoprotective capacities of survivin make it a strong inducer of tumor cell survival in a post-PDT environment.

##### TNF-α

In addition to activating the NF-κB response that stimulates survival, TNF-α is known as a potent trigger of apoptosis *via* the extrinsic pathway as well as necrosis *via* programmed necrosis or necroptosis. When it binds TNF-α, TNFR1 homodimerizes and recruits TRADD and TRAFs to its cytoplasmic domain. In turn, TRADD activates FAS-associated with death domain (FADD) and RIP1, which cleaves procaspase 8 to its active form. Subsequently, caspase 8 cleaves BH3 interacting domain death agonist (BID), yielding truncated BID (tBID) that forms a pore in the mitochondrial membrane and allows cytochrome c leakage. Cytochrome c leakage results in its binding to apoptotic protease activating factor 1 (APAF-1); activation of caspases 9, 3, and 7; and the subsequent activation of the caspase cascade and corollary execution of apoptosis (reviewed in [184]).

Programmed necrosis is the result of RIP1 activation (by *e.g*., TNF-α), which forms an autophosphorylating complex with RIP3. This complex activates mixed lineage kinase domain-like protein that interacts with members of the phosphoglycerate mutase family, culminating in the dephosphorylation of dynamin-related protein 1 and the execution of necrosis [184, 219]. The inhibitor of apoptosis proteins (IAPs) constitute the inhibitors of these cell death pathways, which are also upregulated by the NF-κB-TNF-α signaling loop (Section 3.4.2). IAPs have a plethora of functions, and only a brief summary of the most relevant functions is given here. cIAP1/2 act as ubiquitin ligases for RIP1, thereby inhibiting the apoptotic and necroptotic pathways orchestrated by TNF-α while also stimulating RIP1-mediated IKK activation (reviewed in [220]). Additionally, cIAP1/2 is capable of inhibiting the functions of caspases 3, 7, and 9 and therefore of preventing the execution of apoptosis (reviewed in [221]). cIAP1/2 also inhibits TNF-α signaling by polyubiquitination of NIK and activates JNK and p38^MAPK^ [222, 223] to regulate survival, apoptosis, inflammation, and proliferation, which is covered in greater detail in the context of the ASK1 survival pathway (Section 3.4). As stated previously (Section 3.2.2.2 Survivin), survivin is also an IAP family member that inhibits apoptosis and regulates mitosis (reviewed in [145]).

##### IL-6

One of the most abundant cytokines released by PDT-treated tumor cells is IL-6, which is upregulated by NF-κB and AP-1 transcription factors [224]. IL-6 functions as a proinflammatory cytokine that binds to the IL-6 receptor (IL-6R) expressed predominantly by immune cells and hepatocytes, or to soluble IL-6R (sIL-6R), which is formed *via* alternative splicing of IL-6R mRNA. The IL-6-IL6R and IL-6/sIL-6R complexes can heterodimerize with glycoprotein 130 (gp130) that is ubiquitously expressed by most cell types, including tumor cells [225]. Stimulated gp130 autophosphorylates its intracellular tyrosine kinase domain [225], leading to activation of Janus kinase proteins and the phosphorylation and subsequent nuclear translocation of STAT3 [226]. Moreover, IL-6 triggers proliferation by activating the RAS-MAPK and PI3K-protein kinase B pathways, resulting in the expression of WNT and COX-2 [226]. *Via* these pathways, IL-6 trans-signaling induces the epithelial-mesenchymal transition of tumor cells that promotes invasion, metastasis, and disease progression [227–229]. STAT3 is regarded as the main effector of IL-6 signaling and plays an important role in the survival and proliferation of tumor cells and immune cells [230]. Moreover, STAT3 enhances angiogenic signaling and regulates the production of chemoattractants for neutrophils and macrophages [231]. Upon dimerization, STAT3 binds to interferon (IFN)γ-activated sequence elements to promote survival by upregulating *BCL2L1*, myeloid leukemia cell differentiation protein (*MCL1*), *BIRC4*, and *BIRC5* (survivin) while downregulating *TP53* [231]. Survival is additionally stimulated through upregulation of HSP70, regenerating islet-derived protein IIIβ and γ, trefoil factor 3, as well as the antioxidant enzymes Mn-SOD, ferritin, and catalase (reviewed in [231]). Proliferation is induced *via* STAT3 by upregulation of c-JUN, c-FOS, c-MYC, as well as cyclins D and B that mediate cell cycle progression through the G1/S and S/G2 phases, respectively. STAT3 also promotes angiogenesis by facilitating the production of VEGF, HIF-1α, and basic FGF. Besides its role in tumor (re)growth, STAT3 also prompts the immune system by assisting in the production of a wide array of proinflammatory cytokines and chemokines that includes, but is not limited to, CCL2, CXCL2, IL-1β, IL-1α, TNF-α, and IFNγ (the role of STAT3 in conjunction with NF-κB is comprehensively reviewed in [231]).

##### Matrix metalloproteinases

Remodeling of the tumor microenvironment is essential for cancer progression, and NF-κB stimulates the expression of enzymes that facilitate extracellular matrix remodeling. MMPs are a family of proteins that cleave matrix peptides to facilitate extracellular matrix remodeling, cell migration, and angiogenesis [232]. These proteins are abundantly expressed by tumor cells, tumor-associated fibroblasts, endothelial cells, and tumor-infiltrated immune cells [233]. MMPs also act as signaling molecules that inhibit apoptosis [232]. By contrast, MMPs have been associated with reduced angiogenesis due to the generation of the antiangiogenic compounds angiostatin and endostatin during the degradation of plasminogen (MMPs 2, 3, 7, 9, and 12) and collagen XVIII (MMPs 3, 9, 12, 13, and 20), respectively [234]. The exact role of MMPs in tumor biology and responsiveness to PDT is currently elusive and deserves further context-dependent investigation.

#### Role of the NF-κB pathway in PDT

NF-κB is one of the major transcription factors induced by PDT [194, 195, 235–239], although in some instances NF-κB was also found to be downregulated following PDT, such as in nasopharyngeal carcinoma (hypericin as photosensitizer) and breast cancer cell lines (C-phycocyanin as photosensitizer) [240, 241]. Despite the elusive NF-κB activation mechanism(s) in case of PDT, it is clear that NF-κB activation does occur after PDT on the basis of findings concerning at least two downstream targets of the NF-κB transcription factor, namely COX-2 and survivin. COX-2 mRNA and protein levels as well as COX-2 activity were increased after PDT in a multitude of studies [202, 239, 242–246], albeit COX-2 activity was not necessarily attributed to NF-κB activation [247] but rather to IL-6 or p38^MAPK^ signaling [243, 244, 248]. Similarly, survivin (Section 3.2.2.2 Survivin) was upregulated and phosphorylated after PDT in a number of studies [249–253]. This upregulation was most likely mediated by E2F and STAT3 transcription factors [254], which are indirectly activated by PDT through growth factors (*e.g*., epidermal growth factor (upregulated *via* the ASK1-AP-1 pathway, Section 3.4.2.2 Prolonged downstream effects of ASK1 activation) and VEGF) and cytokines (IL-6) downstream of the HIF-1 and NF-κB pathways (Section 3.2.2). IL-6 functions as a survival factor and also as a regulator of the antitumor immune response after PDT by activating STAT3 and COX-2. Presently, it is unclear whether inhibition of IL-6 signaling by for example blocking AP-1 and/or NF-κB is beneficial or detrimental to tumor response. Several studies have explored the function of IL-6 following PDT, but the investigations have yielded contradictory results. First, expression levels of IL-6 vary depending on the cell line, at least in case of nasopharyngeal cancer cell lines. Whereas CNE-2 cells showed a 13-fold increase in IL-6 mRNA levels compared to untreated cells, HK-1 cells exhibited only a 1.4-fold increase in IL-6 mRNA levels 6 h post-PDT. The effect of IL-6 overexpression on the response to PDT was not investigated [255]. Secondly, the outcomes regarding the prosurvival or prodeath role of IL-6 are conflicting. On the one hand, IL-6 stimulated tumor cell survival and negatively regulated the antitumor immune response in mice bearing Colo26 xenografts [256]. Similarly, IL-6 induction by PDT was associated with cell death inhibition and enhanced tumor growth in human basal cell carcinoma (BCC-1/KMC) cells [247] and mice bearing subdermal Co26 murine colon carcinomas or 4T1 mammary carcinomas [256]. On the other hand, a beneficial effect of IL-6 overexpression for PDT has been reported. Tumor growth in mice was reduced by IL-6 in human prostate cancer (LnCAP) xenografts [257] and human neuroblastoma (WAC2) xenografts [258]. Similarly, mice bearing Lewis lung carcinomas were more susceptible to PDT when the cells overexpressed IL-6 [259]. In a clinical setting, high levels of IL-6 following PDT of cholangiocarcinomas correlated positively with increased tumor mass, indicating that elevated IL-6 levels enhance tumor growth and/or recurrence following PDT [260]. With respect to transcriptional regulation of MMPs after PDT, the AP-1 transcription factors FOS and ATF2 that are activated in the ASK1 survival pathways (Section 3.4.2.1 Acute downstreameffects of ASK1 activation) as well as NF-κB are able to upregulate the expression of MMP1, MMP2, MMP3 [261, 262], and MMP9 [263]. However, the regulation of MMPs following PDT is ambivalent. For example, MMPs 1–3 were upregulated or activated in HK-1 nasopharyngeal carcinoma cells, MCF-7 mammary carcinoma cells and xenografts, in a Walker carcinosarcoma model, and in keratinocyte-associated fibroblasts that were subjected to PDT [240, 264–266]. Furthermore, long-term upregulation of MMP9 but not MMP1, MMP3, MMP7, and MMP12 was observed in actinic keratosis patients treated by PDT [267]. Conversely, several studies have reported the downregulation of MMPs after PDT, including MMP2 and MMP9 in human nasopharyngeal carcinoma, oral cancer, medulloblastoma, and glioma cell lines (HK-1; UP and VB6; MED TE-671; and U87 and GBM6840 cells, respectively) [240, 268–270]. The reduction in MMP2 and MMP9 levels was associated with retarded tumor cell migration [268, 269]. Furthermore, human glioma spheroids treated with PDT exhibited reduced MMP7 and MMP8 levels, a depolarized morphology, and a reduced migration and invasion capacity compared to untreated spheroids [271].

#### Inhibition strategies for NF-κB and its downstream targets

##### Inhibition of NF-κB

Terpenoids are NF-κB inhibitors with a variety of structures and various modes of action [272] (Table 1). The sesquiterpene parthenolide has proven particularly useful for promoting the alkylation of Cys38 in the RelA subunit of NF-κB, thereby preventing DNA binding [137, 272]. Alternatively, panepoxydone is an inhibitor of NF-κB that blocks phosphorylation of IκB by TNF-α without coincident activation of AP-1 in COS-7 monkey kidney cells [138]. However, neither parthenolide nor panepoxydone has been employed in studies combined with PDT. The small molecule Bay 11-7082 is frequently used as a chemical inhibitor of IKK to prevent NF-κB activation both *in vitro* and *in vivo*. This compound effectively inhibited TNF-α-mediated phosphorylation and activation of IKK in lipopolysaccharide-treated murine RAW264.7 macrophages, which translated to impaired phosphorylation and cytosolic retention of NF-κB [139]. However, as shown by Lee *et al*., the main target of Bay 11-7082 does not appear to be IKK since it also inhibits AKT phosphorylation upstream of IKK activation in the TNF-α signaling pathway [139]. The activity of c-FOS and c-JUN was also diminished by Bay 11-7082 in COS-7 cells [139]. Thus, the inhibitory mechanism of Bay 11-7082 on NF-κB stems from its action at sites upstream of IKK rather than direct modulation of NF-κB activity (Table 1).

The precise mechanism notwithstanding, Coupienne *et al*. demonstrated that LN18 human glioblastoma cells were significantly more sensitive to ALA-PDT following incubation with 10 μM of Bay 11-7082 for 30 min prior to PDT [273]. Contrastingly, we have recently demonstrated that siRNA-mediated inhibition of the RelA subunit of NF-κB resulted in reduced susceptibility of murine mammary carcinoma (EMT-6) cells to ZnPC-PDT. While cell viability post-PDT increased in RelA-inhibited cells, the PDT-treated cells released increased levels of TNF-α, CCL2, and IL-6. Accordingly, the supernatant isolated from these cells exerted enhanced immunogenicity on RAW264.7 murine macrophages [274]. In agreement with these results, Chen *et al*. found that Bay 11-7082 and also SP600125, an inhibitor of JNK (Table 1), greatly reduced the amount of apoptotic human Ca9-22 oral cancer cells following ALA-PDT, suggesting that NF-κB and JNK jointly regulate apoptotic signaling following PDT [275]. However, given the multitude of inhibitory effects of Bay 11-7082, it is difficult to ascertain whether the increased or reduced sensitization to PDT is the result of NF-κB inhibition or of impaired AP-1 activity. Rapozzi *et al*. reported that pheophorbide A-PDT in combination with the NF-κB inhibitor dehydroxymethylepoxyquinomicin (DHMEQ, Table 1) [140] promoted cell death in B78-H1 murine amelanotic melanoma cells compared to PDT without DHMEQ [276], which is in support of PDT-induced NF-κB-mediated survival signaling.

Since NF-κB and HIF-1 share a similar activation mechanism following PDT (Sections 3.2.2 and 3.3.2), α-ketoglutarate may serve as an inhibitor of both signaling cascades (Table 1). PHD1 and 3 lose their HIF-1 and NF-κB inhibitory capacity under hypoxic conditions, but the activity of PHDs can be restored by increasing intracellular α-ketoglutarate levels, even under low oxygen tensions [277]. Moreover, the activation pathways of NF-κB and HIF-1 are highly interconnected due to transcriptional upregulation of HIF-1α mRNA by NF-κB and also the HIF-1-mediated production of cytokines, such as TNF-α, that can activate NF-κB. Since hypoxia does not play a major role in the activation of NF-κB [168], NF-κB activation is more likely to result from TNF-α production downstream of the HIF-1 and AP-1 pathways. However, studies in our lab with liposomal zinc phthalocyanine-PDT have shown that incubation of tumor cells with free or liposome-delivered α-ketoglutarate does not enhance PDT efficacy (Broekgaarden, M. et al., Nano Research, in resubmission; Weijer, R. et al., Oncotarget, in resubmission), which is further discussed in Section 3.3.4.

##### Inhibition of COX-2

COX-2 is an important regulator of post-PDT survival [278] insofar as inhibition of COX-2 prior or during PDT has consistently yielded increased tumor cell death after PDT [242, 244, 245, 251, 279–281]. Since COX-2 is under the control of both NF-κB and ATF2, inhibition of NF-κB (with, *e.g*., Bay 11-7085) and also p38α (with, *e.g*., PD169316, SB202190, or SB203580, Table 1) indeed reduced COX-2 protein levels and increased the responsiveness to PDT in human ovarian (HeLa) and bladder cancer (T24) cells as well as radiation-induced mouse fibrosarcoma (RIF-1) cells [202, 239, 244]. In addition, suppression of the AP-1 activators protein kinase C (PKC) and MKK1 and 2 led to decreased COX-2 levels in hypericin-PDT-treated T24 cells and porfimer sodium-PDT-treated RIF-1 cells [202, 239]. These results further attest to the importance of the AP-1 and NF-κB signaling pathways in terms of COX-2 activation and the survival response that ensues after PDT. The most commonly used COX inhibitors are nonsteroidal anti-inflammatory drugs (NSAIDs), which bind to Arg120 of COX-1 and COX-2 to subsequently block the conversion of arachidonic acid to PGH_2_ [142, 282, 283] (Table 1). Some NSAIDs bind only to COX-1 (*e.g*., flurbiprofen), whereas others bind to both COX-1 and COX-2 (*e.g*., naproxen, indomethacin, ibuprofen, and aspirin) [284] or inhibit COX-2 directly, including celecoxib, rofecoxib, nimesulide, diclofenac, meloxicam, and the related compound NS-398 [142, 284, 285]. The latter two groups of inhibitors are suitable for use in PDT because they target COX-2. Accordingly, inhibition of COX-2 prior or during PDT with NSAIDs decreased tumor cell survival in a variety of (tumor) cell lines [242, 245, 251, 279–281, 286], which coincided with a reduction in levels of PGE_2_ [244, 280] and the proangiogenic factors MMP9, TNF-α, IL-1β, IL-10, and VEGF [280]. Moreover, inhibition of COX-2 with NSAIDs caused a reduction in the levels of the antiapoptotic protein survivin [251].

One of the concerns of inhibiting COX-2 activity is that the consequent reduction in cytokine production may abolish the antitumor immune response necessary for long-term protection against tumor recurrence [169] and removal of residual or non-PDT damaged tumor cells in immunocompetent hosts [83, 84]. However, blocking of COX-2 with celecoxib, NS-398, or nimesulide showed considerably increased survival of immunodeficient mice in which various tumor cell lines were xenografted [242, 245, 251, 280]. Thus, the inhibition of COX-2 activity with NSAIDs could be a valuable intervention strategy for PDT to reduce tumor cell survival and potentially reduce the proangiogenic effects induced by PGE_2_.

##### Inhibition of survivin

Inhibition of survivin, which is upregulated by activation of NF-κB following PDT (Section 3.2.2.2 Survivin), may reduce antiapoptotic signaling and therefore could result in increased PDT efficacy. Several different compounds that inhibit survivin are available that either block upstream activators such as HSP90 (17-AAG) and STAT3 (STA-21 [143] or WP1066 [144]) or inhibit survivin directly *via* antisense RNA interference (LY218130B) and/or transcriptional repression (YM155 and EM1421) [145] (Table 1), although the specificity of the latter compounds may not be restricted to survivin [287].

Some investigations studying the inhibition of survivin during PDT have employed geldanamycin (17-AAG) to inhibit HSP90-induced survivin expression [250, 252], celecoxib or 2,5-dimethyl celecoxib [251] for direct inhibition of survivin (although the mechanism by which these compounds inhibit survivin remains elusive), or have applied gene knockdown strategies [249]. Regardless of the inhibition strategy, all these studies point toward an increased tumoricidal effect of survivin inhibition during PDT, making survivin an important target for PDT enhancement strategies.

##### Inhibition of IL-6

Unequivocal evidence for the prosurvival role of IL-6 in PDT-subjected tumor cells is lacking since both beneficial and detrimental effects of IL-6 signaling in terms of cell survival have been observed after PDT (Section 3.2.2.4 IL-6). Although the use of IL-6 inhibitors has not been explored in PDT research, cancer-related studies in which IL-6 signaling was inhibited may provide clues as to the potential (neo)adjuvant efficacy of IL-6 inhibitors for the enhancement of PDT. A specific blocker of IL-6/sIL-6R transactivation has been developed by fusing the extracellular domain of human gp130 to a human IgG1 antibody (sgp130Fc, Table 1). The molecule was shown to effectively block IL-6 signaling in mouse and rat models of autoimmune disease (reviewed in [146] and [288]). For example, sgp130Fc significantly prevented disease progression in inflammation-associated mouse cancer models. Thus, blocking of IL-6 transactivation with sgp130Fc after PDT could increase the therapeutic potential and may be instrumental in elucidating the role of the IL-6 signaling pathway in tumor cell survival.

##### Inhibition of matrix metalloproteinases

Inhibitors for MMPs are readily available, and most agents inhibit multiple MMP isoforms. This is particularly important since the antitumor effects of MMP inhibitors are not confined to a single isozyme but are mediated by, *e.g*., MMP3, MMP8, and MMP12 [232]. Ferrario *et al*. investigated the broad spectrum MMP inhibitor prinomastat [147] (Table 1) in combination with PDT in mouse BA mammary carcinoma xenografts [289] after observing increased levels of MMP2 and MMP9 expression after porfimer sodium-PDT. Long-term cures were found in 46 % of mice treated with prinomastat and PDT *versus* only 20 % in mice treated with PDT alone, although the enzymatic activity in the presence of prinomastat was not assayed. Accordingly, the inhibition of MMPs during PDT holds potential for the enhancement of therapeutic efficacy. Despite the positive results, caution should be exercised when designing an MMP inhibitor-based combinatorial treatment in light of the variable regulation of different MMP isozymes and their ambivalent biological effects (tumor suppressing and tumor promoting). For example, wound healing relies on MMPs, and it is possible that pharmacological inhibition may interfere with the recovery of PDT-treated tissues.

#### Concluding remarks

The contribution of NF-κB to the cell survival response appears to be well-established based on the studies that have demonstrated NF-κB activation following PDT (Section 3.2.3). At least three possible mechanisms are responsible for NF-κB activation after PDT (Section 3.2.1) and pharmacological interventions in the NF-κB survival pathway are possible to improve PDT outcomes (Section 3.2.4). However, such interventions may present a therapeutic quagmire. On the one hand, the downstream targets of NF-κB are instrumental for tumor cell survival following PDT, such as COX-2 and survivin, of which the inhibition results in increased tumor cell death and better tumor control (sections 3.2.4.2 Inhibition of COX-2 and 3.2.4.3 Inhibition of survivin). On the other hand, many proinflammatory cytokines are upregulated by NF-κB that can attract cells of the innate and adaptive immune system to mediate an antitumor immune response. Interfering with the capability of the treated cancer cells to produce a variety of cytokines and chemokines may therefore inhibit the antitumor immune response and reduce long-term therapeutic efficacy. In contrast to the postulations, it was recently shown that inhibition of NF-κB resulted in increased cytokine release and immunogenicity of PDT-treated tumor cells *in vitro* [274] and suggested that NF-κB may not be a suitable target for pharmacological inhibition in conjunction with PDT. These contrasting results demonstrate that further research on the *in vitro* and *in vitro* consequences is pivotal to understand the complex functions of NF-κB in a post-PDT tumor microenvironment.

### The HIF-1 pathway

Tumor growth frequently leads to hypoxia since the tumor tissue tends to outgrow its immature blood supply, as a result of which a hypoxia-induced inflammatory response is triggered to stimulate angiogenesis and increase metastasis. Tumor cells cope with mildly hypoxic conditions by constitutively activating HIF-1, leading to the transcription of genes involved in anaerobic metabolism, inflammation, and antioxidant responses [290]. Under conditions of acute severe hypoxia or anoxia, tumor cells hyperactivate HIF-1 and its downstream responses for purposes of survival (Broekgaarden, M. et al., Nano Research, in resubmission; Weijer, R. et al., Oncotarget, in resubmission). HIF-1 activation has been observed in many PDT studies, and HIF-1 has been accepted as one of the main molecular effectors induced by PDT [246, 250, 291–294]. The remainder of this section will address the four main activation mechanisms of HIF-1 (Section 3.3.1) and the most important downstream effects that may play a role in tumor cell survival post-PDT (Section 3.3.2). Evidence for its activation after PDT is addressed in Section 3.3.3, and the potential HIF-1 intervention strategies to enhance PDT efficacy are discussed in Section 3.3.4.

#### Activation mechanisms of HIF-1

**Fig. 5:**
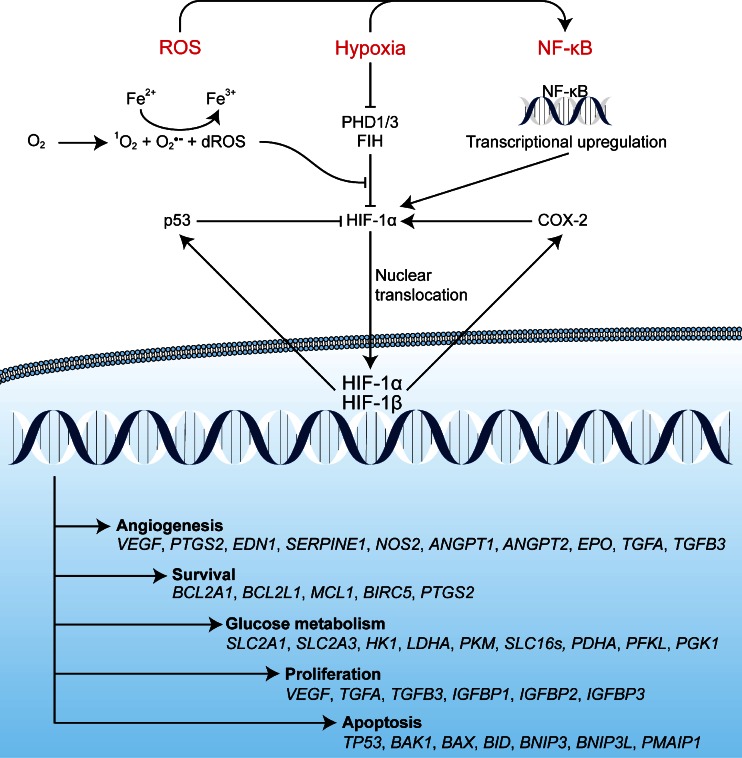
Activation of HIF-1α after PDT is mediated by several pathways. PDT-induced hypoxia due to immediate O_2_ depletion as well as vascular shutdown prevents HIF-1α hydroxylation by PHDs and FIH, which is an O_2_-dependent process. Furthermore, ROS-mediated oxidation of Fe^2+^ in the catalytic center of PHDs and FIH disables the enzymatic activity of these proteins. Both events lead to HIF-1α stabilization, translocation from the cytosol to the nucleus, complexation with HIF-1β, and transcriptional upregulation of numerous target genes containing an HRE in the promoter region. HIF-1α transcription is also upregulated by PDT-activated NF-κB, which increases HIF-1α protein levels. A negative feedback loop for HIF-1α exists *via* the upregulation of p53 by activated HIF-1, which targets HIF-1α for proteasomal degradation in the presence of DNA damage. A positive feedback loop exists *via* the upregulation of COX-2 by activated HIF-1 and NF-κB. COX-2 is involved in the production of PGE_2_ that plays a role in the transactivation of HIF-1α. After nuclear translocation, dimerization, and DNA binding to HRE sequences, HIF-1 transcription factors facilitate the upregulation of a plethora of genes involved in angiogenesis, survival, glucose metabolism, proliferation, and apoptosis. Other pathways are affected as well, but only those most relevant for PDT are depicted

The HIF-1 transcription factor is a basic helix-loop-helix (bHLH) heterodimeric protein composed of an α subunit (HIF-1α or HIF-2α) and a β subunit (HIF-1β) subunit [295]. HIF-1α is constantly transcribed but retained in the cytosol and rapidly degraded under normophysiological conditions. HIF-1β is constitutively expressed in the nucleus, where it is separated from its dimerization partner HIF-1α in the cytosol and thus kept inactive. Upon stabilization, HIF-1α translocates to the nucleus, dimerizes with HIF-1β, and binds DNA at hypoxia responsive elements (HREs) to initiate target gene expression [296, 297]. The effects of HIF-1 activation are profound, since over 500 genes are known to be a direct target of HIF-1. Moreover, HIF-1 is involved in chromatin remodeling complexes and microRNA expression that regulate gene expression at an epigenetic level [298–301]. There are at least four different mechanisms by which HIF-1α may become activated after PDT, namely hypoxia, ROS, NF-κB, and COX-2. The pathways are summarized in Fig. 5.

##### HIF-1 activation by hypoxia

HIF-1α acts as an oxygen sensor in that it is constantly targeted for proteasomal degradation under normoxic conditions as a result of hydroxylation and subsequent polyubiquitination [295, 297, 302–305]. Hydroxylation of HIF-1α by PHD2/3 and FIH leads to HIF-1α recognition and binding by VHL proteins, which act as a scaffold for E3 ubiquitin ligase that polyubiquitinates HIF-1α as a signal for proteasomal degradation [306, 307]. During hypoxia, which occurs after PDT (Section 2.2.2), HIF-1α hydroxylation by PHDs and FIH ceases because the hydroxylation reaction requires O_2_ [308]. This causes HIF-1α to become stabilized, move to the nucleus, complex with HIF-1β, and activate gene transcription through HREs.

##### HIF-1 activation by ROS

HIF-1α stabilization by hypoxia-mediated PHD and FIH inactivation can also proceed through ROS-mediated deactivation of PHDs and FIH in a manner that is not necessarily dependent on intracellular oxygen tension [309–311]. PHDs and FIH require Fe^2+^ as cofactor in their conversion of α-ketoglutarate, O_2_, and proline to succinate, CO_2_, and hydroxyproline, respectively. It should be noted that succinate is an important electron donor in the citric acid cycle [312]. Oxygen radicals, which are abundantly produced during PDT (Section 2.2.1), are able to oxidize Fe^2+^ to Fe^3+^, thereby inhibiting the enzymatic activity of PHDs and FIH and reducing hydroxylation-mediated degradation of HIF-1α, even under normoxic conditions [313]. The ROS-driven activation of HIF-1 may also be significant under hypoxic conditions. Hypoxia induces mitochondrial ROS release that was shown to be responsible for an initial burst of HIF-1 activation, whereas hypoxia-driven, NADPH-oxidase-induced ROS induced a second burst of HIF-1 activation in human HS29-4T melanoma cells [314]. Thus, the activation mechanisms of HIF-1 by ROS and hypoxia appear to be interrelated.

##### HIF-1 activation by NF-κB

In addition to modulation by ROS and hypoxia, HIF-1α expression is at least partially under the control of NF-κB since the promoter of the *HIF1A* gene contains an NF-κB binding site [197]. Cultured pulmonary artery smooth muscle cells incubated with H_2_O_2_ under normoxic conditions exhibited NF-κB activation that was responsible for transcriptional upregulation of HIF-1α and the HIF-1 target gene plasminogen activator inhibitor 1 (PAI1) [315]. Given that NF-κB is activated by inflammation, hypoxia, and ROS (Section 3.4.1), transcriptional upregulation of *HIF1A* by NF-κB and subsequent translation to a functional unit may be one of the most important regulatory mechanisms that influences HIF-1 signaling.

##### HIF-1 activation by COX-2

The intricate relationship between NF-κB and HIF-1 signaling is further exemplified through COX-2. In addition to transcriptional regulation by NF-κB, the *PTGS2* gene that encodes COX-2 contains four HRE sequences in its promoter region, explaining its upregulation by HIF-1 under hypoxic or prooxidative conditions [201]. Interestingly, COX-2 amplifies the HIF-1 pathway. COX-2 facilitates the production of PGE_2_ (Section 3.2.2.1 COX-2), which can subsequently stimulate the transcriptional activity of the HIF-1 complex [201]. As such, there is a positive feedback loop for COX-2 and HIF-1 activity, although the exact molecular interactions have not been fully elucidated.

#### Downstream effects of the HIF-1 pathway

When the growth of the tumor parenchyma is more extensive than the formation of new blood vessels, the deficiency in oxygen supply will trigger HIF-1 activation that in turn signals a metabolic switch to glycolysis and a consequent decrease in oxygen demand. The consequent decrease in O_2_ consumption expands the area of O_2_ availability within the tumor, allowing distally situated tumor cells (relative to the vasculature) to proliferate, which benefits tumor growth as a whole [297]. Inasmuch as persistent hypoxia can only be resolved by the formation of new blood vessels, HIF-1 signaling is programmed to stimulate angiogenesis [316] (Fig. 5). The vascularization of a tumor requires degradation of the extracellular matrix to enable vessel sprouting, migration, and maturation of mesenchymal cells into endothelial cells; tube formation; and pericyte recruitment to endothelialize the newly formed lumens (reviewed in [317]). Therefore, a hypoxic tumor microenvironment and the HIF-1 transcription factor are important mediators of cell survival and tumor regrowth following therapy.

With respect to glucose metabolism, tumor cells and tumor-associated cells become less dependent on oxygen during hypoxia by reducing oxidative phosphorylation and increasing anaerobic respiration (*i.e*., glycolysis; Warburg effect) [318]. HIF-1 is instrumental in this transformation by initiating the transcription of genes involved in glucose metabolism. The target gene products include glucose transferases 1 and 3 (GLUT1/3, *SLC1A1*/*3*), hexokinase (HK, *HK1*), lactate dehydrogenase A (LDHA), monocarboxylate transporters (MCTs, *SLC16As*), pyruvate dehydrogenase (PDH), pyruvate kinase (PKM), phosphofructokinase L (PFKL), and phosphoglycerate kinase I (PGK1) (reviewed in [297] and [296]) (Fig. 5). Despite the prevailing state of hyponutrition as a result of PDT-induced vascular shutdown, residual viable tumor cells may scavenge glucose from the tumor microenvironment to support anaerobic respiration. This glucose may have been released from tumor cells immediately killed by PDT to support anaerobic respiration. Intratumoral angiogenesis, endothelial cell proliferation, and matrix and vascular remodeling are modulated by HIF-1 *via* upregulation of *VEGF*, endothelin 1 (*EDN1*), plasminogen activator inhibitor 1 (PAI1, *SERPINE1*), (inducible) nitric oxide synthase 2 (*NOS2*), angiopoietin (*ANGPT*) *1* and *2*, erythropoietin (*EPO*), and transforming growth factor (TGF)-β3 (*TGFB3*) [299, 319] (Fig. 5). Proliferation of tumor and tumor-associated cells is stimulated by HIF-1 through the induction of genes encoding insulin-like growth factor (IGF) 2 as well as IGF binding proteins 1, 2, and 3; TGF-α and TGF-β3; and VEGF [296, 297] (Fig. 5). In this process, COX-2, which is a target gene of HIF-1 (Section 3.3.1.4 HIF-1 activation by COX-2), orchestrates a positive feedback loop that reinforces the activity of both COX-2 and HIF-1 [201] (Fig. 5). PGE_2_ is produced by COX-2 and enhances *HIF1A* transcription and induction of HIF-1, which subsequently binds the COX-2 promoter to upregulate its expression [201]. Taken altogether, HIF-1 potentiates numerous critical biological responses to PDT that revolve around tumor cell survival and enables cells to cope with and recover from the damage caused by PDT. Lastly, HIF-1 has been shown to have notable effects on cell death pathways. In addition to transcriptionally upregulating survivin (*BIRC5*) (Section 3.2.2.2 Survivin) and HO-1 (Section 3.1.2), HIF-1 regulates prosurvival proteins of the BCL2 family (BCL2 (*BCL2A1*), BCL-XL (*BCL2L1*), BID, and MCL-1 (*MCL1*)) (Fig. 5), although proapoptotic members of the same family have also been reported to be upregulated by HIF-1, including BCL2-homologous antagonist killer (BAK), BAX, BCL2/adenovirus E1B 19 kDa protein-interacting protein 3 (*BNIP3*), BNIP3 ligand (*BNIP3L*), and NOXA (phorbol-12-myristate-13-acetate-induced protein 1, *PMAIP1*) [320]. However, HIF-1-mediated induction of BNIP3 and BNIP3L may also be essential for hypoxia-driven cytoprotective autophagy and facilitate hypoxic survival, at least in human prostate cancer (PC-3) cells [321]. Furthermore, HIF-1 has been implicated in the stabilization of tumor suppressor protein p53 [322], which promotes apoptosis upon oncogenic stress and negatively regulates HIF-1α stability (Fig. 5) [323]. Although HIF-1 predominantly stimulates survival through various biological processes, proapoptotic signaling may also occur in the presence of DNA damage *via* p53-mediated activation of proapoptotic BCL2-family members that are upregulated by HIF-1.

#### Role of the HIF-1 pathway in PDT

Although HIF-1 is considered an important transcription factor in the context of PDT [17], very few studies have investigated HIF-1 activity following PDT. Chemical induction of HIF-1 by preincubating human Het-1 esophageal cells with 500 μM CoCl_2_ desensitized cells to ALA-PDT [324]. Mitra *et al*. demonstrated that HIF-1 is activated by porfimer sodium-PDT in murine breast cancer (EMT-6) cells transfected with a gene encoding green fluorescent protein (GFP) under the control of a promoter sequence with five HREs [293]. The expression of GFP after PDT occurred under normoxic conditions, underscoring the relevance of ROS-mediated activation of HIF-1 in the absence of hypoxia (Sections 3.3.1.2 HIF-1 activation by ROS and 3.3.1.3 HIF-1 activation by NF-κB). The authors argued that PGE_2_ synthesized by COX-2 (Section 3.3.1.4 HIF-1 activation by COX-2) may be an important mediator of HIF-1 activity, although no corroborative evidence was obtained in COX-2 inhibition experiments [293]. The technical difficulties in studying HIF-1 in an *in vitro* PDT setting result from the requirement for hypoxic culture conditions and the short half-life of HIF-1α under normoxic conditions (5–8 min) [325]. Despite these difficulties, Krieg *et al*. showed increased HIF-1α protein expression following ALA-PDT in UROtsa, RT112, and J82 (but not RT4) human bladder cancer cells under normoxic conditions using reversed phase protein arrays [292]. Stabilization and activation of HIF-1 under hypoxic conditions was recently demonstrated in human epidermoid carcinoma (A431) and human extrahepatic cholangiocarcinoma (Sk-Cha1) cells after PDT with liposomal zinc phthalocyanine. In line with HIF-1α stabilization, *VEGF*, *PTGS2*, and *HMOX*-*1* mRNA were upregulated to a greater extent after PDT than in untreated hypoxic cells (Broekgaarden, M. et al., Nano Research, in resubmission; Weijer, R. et al., Oncotarget, in resubmission). Additional evidence for the prominent role of HIF-1 in PDT was provided in a mouse model of Kaposi’s sarcoma using porfimer sodium-PDT. Tumors collected 1 h after PDT exhibited increased HIF-1α protein levels compared to untreated tumors. The HIF-1α protein levels in PDT-treated tumors were comparable to those in tumors of which the blood supply had been clamped for 30 min [326]. Similar results regarding HIF-1 activation were obtained in human nasopharyngeal carcinoma (CNE-2) xenografts in mice that had been subjected to hypericin-PDT [246] and in rat chorioretinal tissue treated with verteporfin-PDT [294]. The increased mRNA expression and protein levels of HIF-1α were associated with increased protein levels of VEGF, as was demonstrated in a murine model of mammary (BA) carcinoma treated with porfimer sodium-PDT [291], indicating that post-PDT HIF-1 signaling induces angiogenic remodeling of the affected tissue (Section 3.3.2 and Fig. 5). Moreover, a clinical study by Koukourakis *et al*. revealed that esophageal tumors with high intratumoral protein levels of HIF-1 were more resistant to PDT compared to tumors with low HIF-1 protein levels [327], attesting to the involvement of HIF-1-mediated survival pathways following PDT (Section 3.3.2 and Fig. 5). Increased HIF-1α protein levels were also observed in mouse porfimer sodium-PDT-treated murine BA mammary carcinoma tumors, but this was not reported for porfimer sodium-PDT-treated BA cells *in vitro* [250].

#### Inhibition strategies for HIF-1 and its downstream targets

Due to the importance of HIF-1 in tumor survival, therapeutic interventions for cancer encompass the inhibition of HIF-1 [290]. However, most HIF-1 inhibitors are rather unspecific and also target the upstream modulators of HIF-1α protein synthesis, of which imatinib (an inhibitor of breakpoint cluster region protein (BCR)-ABL [328]), gefitinib, erlotinib, and cetuximab (an inhibitor of EGFR [329]), and everolimus (an inhibitor of mTOR [330]) are well-known examples [290] (Table 1). Another combination strategy is to interfere with the stabilization of HIF-1 by inhibition of chaperone binding using geldanamycin (an inhibitor of HSP90 [331]) or increasing the affinity for natural inhibitors of HIF-1 (*e.g*., amphothericin B [148]) (Table 1). Interfering with HIF-1 DNA binding is another approach to reduce HIF-1 signaling. For example, echinomycin competes with HIF-1 to bind to HREs and can therefore be used to reduce transcriptional activity of HIF-1 [149] (Table 1). As mentioned previously, these inhibitors are rather unspecific, which may be valuable in the development of a combinatorial cancer therapy. However, a more specific inhibitor of HIF-1 would be desirable when investigating the mechanism of HIF-1 on tumor cell survival following PDT.

α-Ketoglutarate may be a useful drug as a specific inhibitor of HIF-1 (Table 1). Under normophysiological conditions, PHDs are the major inhibitors of HIF-1 activity during normoxia but are rendered dysfunctional during hypoxia [332] (Section 3.3.1 and Fig. 5). The endogenous molecule α-ketoglutarate is a selective PHD substrate and agonist [312], and it is able to reactivate PHDs to inhibit HIF-1 regardless of intracellular oxygen tension [141]. Under normoxic conditions, PHDs facilitate the conversion of α-ketoglutarate and oxygen to succinate and carbon dioxide, respectively, but also transfer oxygen to prolyl residues in the HIF-1α oxygen-dependent degradation domain (ODD) [312]. Increasing the activity of PHDs after PDT with α-ketoglutarate may therefore render cells less susceptible to HIF-1-mediated survival. Studies by Mackenzie *et al*. have shown that, despite hypoxia, the activity of PHD2 and 3 and the concurrent destabilization of HIF-1 in various tumor cell lines and murine xenografts could be induced by the administration of α-ketoglutarate esters (esterification allows passage through the membrane into the cell) [141]. The inhibition of HIF-1 by α-ketoglutarate was associated with decreased tumor growth and increased apoptosis [277, 333]. Based on these investigations, HIF-1 inhibition by α-ketoglutarate may be a valuable strategy in potentiating the effects of PDT. However, recent studies by our group revealed that α-ketoglutarate did not increase the efficacy of PDT, but rather reduced PDT-induced oxidative stress as measured 4 h post-PDT in A431 cells. It was hypothesized that α-ketoglutarate was used as an energy source to fuel antioxidant responses or that it functions as an antioxidant itself and thus is not a suitable agent to enhance the PDT response (Broekgaarden, M. et al., Nano Research, in resubmission; Weijer, R. et al., Oncotarget, in resubmission).

Another nonspecific inhibitor of HIF-1 is the naturally occurring diphenolic compound curcumin (Table 1), which was found to promote proteasomal degradation of HIF-1α and HIF-1β (also known as aryl hydrocarbon receptor nuclear translocator, ARNT) [150, 334, 335]. Moreover, Choi *et al*. [150] showed that HIF-1 activity was reduced as a result of curcumin-stimulated oxidation and proteasomal degradation of HIF-1β. The authors further showed that the inhibition of HIF-1β was dependent on oxidation, as addition of the antioxidant N-acetylcysteine prevented HIF-1β inactivation by curcumin. However, it should be noted that curcumin exerts many other mainly cytostatic/toxic effects on tumor cells, including the inhibition of EGFR tyrosine kinase activity and downstream signaling; inhibition of protein kinase C, COX-2, and NF-κB; and induction of DNA damage [336]. These effects tend to increase PDT efficacy, as exemplified by the finding that curcumin increases ROS production, mitochondrial membrane permeabilization, mitochondrial cytochrome c release, and caspase activation after porfimer sodium-PDT, which exacerbated cell death in human head and neck cancer (AMC-NH3) cells [337]. In contrast, curcumin is also a potent antioxidant [336], a property that was found to reduce the extent of ROS production and thereby the degree of cell death in A431 human epidermoid carcinoma cells treated with rose bengal-PDT [338].

A recently discovered and rather specific inhibitor of HIF-1 activity is acriflavine (Table 1), which inhibits HIF-1α/HIF-1β and HIF-2α/HIF-1β dimerization by binding the Per-Arnt-Sim B domain of HIF-1α and HIF-2α [151]. The binding of acriflavine to HIF-1 blocks DNA binding and reduces transcriptional activity, tumorigenicity, and angiogenic signaling, as was demonstrated in xenotransplanted human prostate (PC-3) and hepatoma (Hep3B) tumors in mice [151]. Results from our lab confirm the feasibility of employing acriflavine to improve PDT efficacy, as the sensitivity of A431 cells to PDT increased as a result of reduced glycolytic activity; downregulation of the HIF-1 target genes *VEGF*, *PTGS2*, and *PAI1*; and upregulation of *EDN1*. Taken together, these results illustrate the importance of HIF-1 in the protection of tumor cells from PDT (Broekgaarden, M. et al., Nano Research, in resubmission; Weijer, R. et al., Oncotarget, in resubmission).

#### Concluding remarks

The HIF-1 transcription factor is essential for the survival of cells in a hypoxic environment and under conditions of acute stress, such as oxidative stress induced by PDT. The survival signaling manifests itself through direct intracellular events (*e.g*., by the production of prosurvival proteins and the shift to oxygen-independent metabolism) as well as through tissue processes (*e.g*., angiogenesis and proliferation) (Section 3.3.2). In case of PDT, it has been shown that HIF-1 activation causes the release of proangiogenic factors (Section 3.3.3).

Since malignant tumors proliferate faster than the rate of neovascularization, most tumors are hypoxic in nature and constitutively activate HIF-1 [316]. Poorly vascularized tumors may therefore be more resistant against PDT due to hypoxic preconditioning (in addition to the suboptimal accumulation of systemically administered photosensitizer molecules as a result of the poor blood supply). Tumors that overexpress HIF-1 are less sensitive to therapy and are associated with poor survival in patients. Accordingly, the coadministration of HIF-1 inhibitors as neoadjuvants increases the efficacy of PDT, as has been demonstrated in several studies (Section 3.3.4).

### The ASK1 pathway

The immediate early stress response is a mechanism in cells that encompasses the rapid transcription and translation of a set of genes coding for protein products that enable cells to adequately adapt to extra- or intracellular stress. Although the exact activation trigger fueling this response is somewhat elusive in relation to PDT, this section reviews the activation of ASK1 in response to generic oxidative stress, similar to that induced by PDT, and to TNF-α signaling (Section 3.4.1). ASK1 relays its signal *via* MAPKs (JNK and p38^MAPK^) to the AP-1 transcription factor family (Section  3.4.2.1.1 JNK and p38 proteins) that is responsible for the rapid induction of immediate early gene transcription. As a whole, the ASK1 signaling pathway exerts both cytoprotective as well as cytodestructive effects, depending on the balance between the activation of the ASK1 pathway and the NF-κB-TNF-α pathway that seem to chiefly govern cell fate (Section 3.2). The available literature on the participation of the ASK1 pathway in the post-PDT response (Section 3.4.5) and inhibition of MAPK activity (Section 3.4.4) are summarized, and possible inhibition strategies for this survival pathway are proposed.

#### Activation mechanisms of ASK1

##### ASK1 activation by ROS

**Fig. 6:**
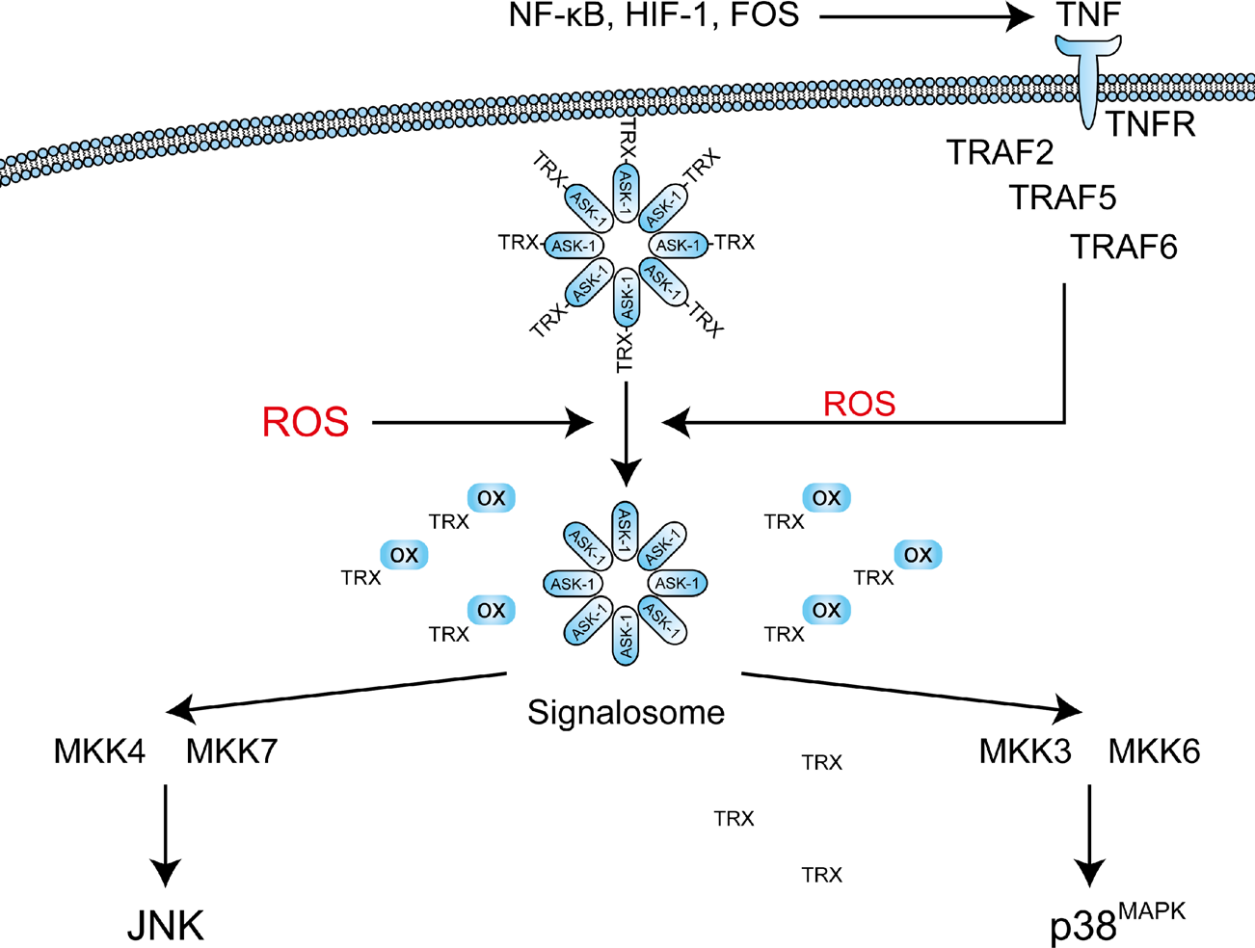
Activation mechanisms of the ASK1 signaling pathway leading to JNK and p38^MAPK^ phosphorylation. ROS can directly or indirectly (*via* GSH) oxidize the TRX subunits (TRX-ox) of the inactivated signalosome complex. Upon oxidation and subsequent dissociation of TRX, the ASK1 heteromer autophosphorylates and initiates downstream signaling. MKK4/7 are kinases responsible for the activation of JNK proteins, whereas MKK3/6 phosphorylate and activate the p38^MAPK^s. Downstream of the NF-κB, HIF-1, and FOS pathways, tumor cells and immune cells produce TNF-α. TNF-α binds the TNFR, which activates intracellular TRAF. These TRAFs stimulate the production of mitochondrial ROS and NADPH oxidase 1-derived ROS that stimulate the dissociation of TRX from the signalosome, but also bind TRX to prevent reassociation of TRX with ASK1

The activation of JNK, p38^MAPK^, and AP-1 transcription factors following oxidative stress is preceded by the activation of the mitogen-activated protein kinase kinase kinase ASK1 [339]. ASK1 forms homo-oligomers in its inactive state, comprising a complex that is referred to as the signalosome, in which multiple ASK1 proteins are bound at their C-terminal coiled-coil domains [340]. Thioredoxin (TRX) binds ASK1 subunits of the signalosome that shield the N-terminal transactivation domain, thereby inhibiting autophosphorylation of threonine (Thr) 845 that is required for signalosome activation [341]. Under oxidative stress (*e.g*., after TNF-α-induced ROS formation), ROS (and oxidized substrates such as proteins and GSSG) mediate the oxidation of TRX [342]. TRX is oxidized at cysteine residues in the active site, leading to its dissociation from the signalosome, subsequent autophosphorylation of ASK1 subunits, and activation of the complex [339, 341, 343] (Fig. 6). Activated ASK1 phosphorylates MAP kinase kinases (MKK3), MKK4, MKK6, and MKK7 at conserved residues within the kinase domain, leading to their activation [344, 345]. MKK4 and MKK7 phosphorylate and activate JNK at Thr183 and Thr185, whereas MKK3 and MKK6 phosphorylate and activate the different p38^MAPK^ isoforms (Section 3.4.2.1 Acute downstreameffects of ASK1 activation) at Thr180 and Tyr182 [346, 347]. In addition to direct activation *via* oxidized TRX, ASK1 signaling may be enhanced *via* paracrine signaling through TNF-α, as is described in the following section.

##### ASK1 activation by TNF-α

The ASK1 pathway is also stimulated by TNF-α (Fig. 6), which is expressed as a result of PDT-activated AP-1 and NF-κB transcription factors, and TNF-α is a potent inducer of apoptosis and programmed necrosis (reviewed in [184]). TNF-α binds to the TNFR, which in turn mobilizes TRAFs 2, 5, and 6 to bind and activate the ASK1 signalosome [340]. ASK1 activation can also be triggered by TNF-α signaling alone [340] as a result of TNF-α-mediated production of ROS by mitochondria and/or NADPH oxidase 1 [348, 349]. ROS production by TNF-α likely involves TRAF2 [350].

#### Downstream effects of ASK1 activation

##### Acute downstream effects of ASK1 activation

**Fig. 7:**
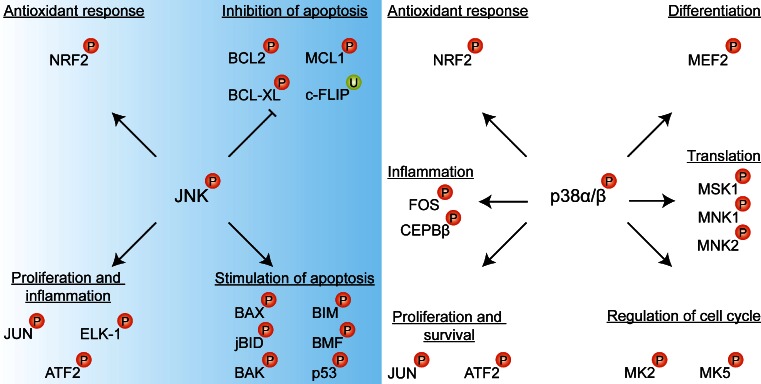
JNK1 exerts kinase activity on several transcription factors and BCL2 family proteins through phosphorylation (*P*) or ubiquitination (*U*). Proliferation and inflammation are induced by phosphorylating members of the JUN and ATF2 protein family, as well as ELK-1. The NRF2 antioxidant response is triggered *via* phosphorylation of NRF2 at Ser40. Apoptosis is stimulated *via* the phosphorylation of BAX, BAK, BIM, BID, and BMF proteins as well as *via* p53 activation. Antiapoptotic proteins are also phosphorylated (and inactivated) by JNK1, which include BCL2, BCL-XL, and MCL-1, or ubiquitinated (c-FLIP). The kinase functions of p38α/β entail a similar effect on the NRF2 antioxidant response pathway as reported for JNK1. Inflammation is stimulated *via* phosphorylation of the AP-1 transcription factors of the FOS and C/EBP protein family. Proliferation and survival are promoted *via* activation of JUN and ATF2. Translation of newly transcribed genes is facilitated by phosphorylation of MSK1, MNK1, and MNK2. Differentiation is mediated *via* MEF2 activation by p38α/β. Additionally, p38α/β regulate the cell cycle by phosphorylation of MK2 and MK5

**Fig. 8:**
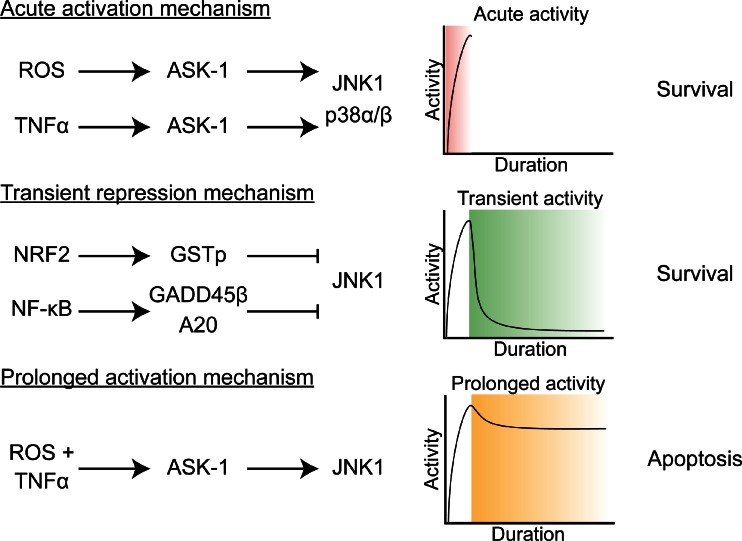
The ambivalent effects of the ASK1 pathway are dictated by the cross-talk between various pathways and the prevailing biochemical conditions. The primary activation mechanism of the ASK1 pathway by PDT emanates from oxidative stress or TNF-α signaling, leading to the acute, survival-promoting activity of JNK1 and p38α/β. Subsequently, downstream of the NRF2 and the NF-κB pathways, negative regulators of JNK1 and p38α/β are produced/activated that modulate the transient activation pattern of these kinases and thus promote cell survival. Whenever ROS and TNF-α signaling occur simultaneously, or whenever these stress signals endure, prolonged JNK1 and p38α/β activation promotes apoptosis

Although the exact role of ASK1 itself is underinvestigated in the context of PDT, the ASK1 signaling cascade has been implicated in both increasing cell death and increasing cell survival in response to ROS or TNF-α. Whereas a transient activation stimulus (*i.e*., short-lasting or mild oxidative stress) encourages survival and proliferation [351], a prolonged activating stimulus (*i.e*., severe and prolonged oxidative stress or prolonged TNF-α/TNFR signaling) promotes growth arrest and apoptosis [352] (Fig. 8). Since the MAPKs JNK and p38^MAPK^ can regulate both survival (*via* a plethora of AP-1-like transcription factors) and cause cell death (*via* the activation of proapoptotic BCL2 proteins), we postulate that these kinases are critical for this cell survival/death switch or checkpoint, as is discussed below and summarized in Fig. 7.

##### JNK and p38 proteins

There are three JNK isozymes, namely JNK1, JNK2, and JNK3, whereby JNK3 is mainly expressed in neural tissue. JNK mRNA is prone to alternative splicing, giving rise to over 10 different isoforms, which explains the diverse biological functions in various tissues [353, 354]. Whereas JNK1 was found to be the most important inducer of downstream signaling and JNK2 is an antagonist that competes with JNK1 for ligand binding [355], a more activating role of JNK2 in downstream signaling induction has also been described [356]. Since most inhibition strategies for JNK signaling were performed with JNK1 inhibitors, the focus will be on this specific isozyme of the JNK family. Although there are four isoforms of p38^MAPK^ proteins, namely p38α, p38β, p38δ, and p38γ, the functions of p38α and p38β are most extensively described [354]. Many inhibition strategies regarding p38^MAPK^ and downstream signaling events were performed with inhibitors for p38α and p38β, so the main focus for the remainder of this review will be on these specific isozymes of the p38^MAPK^ family.

##### AP-1 transcription factors

JNK1 and p38α/β activate a multitude of AP-1-type transcription factors. The AP-1 transcription factor family consists of a large variety of dimers composed of basic leucine zipper domain (bZIP) proteins from the JUN subfamily (JUN, JUNB, and JUND), the ATF2 subfamily (ATF2 and cAMP response element binding, CREB), and the FOS subfamily (FOS, FOSB, FOS-related antigen 1 (FRA1), and FRA2). Another subfamily exists, the MAF subfamily [262], but the function of these proteins in the cellular response to PDT or oxidative stress remains largely unexplored.

As addressed above, JNK1 specifically phosphorylates and activates AP-1 proteins from the JUN and ATF2 subfamilies as well as the non-AP-1 transcription factor ETS domain-containing protein (ELK-1). Other transcription factors such as NRF2 and p53 are phosphorylated and activated by JNK1. p38α/β also phosphorylates JUN and ATF2 proteins and also functions as a kinase for FOS proteins. Moreover, p38α/β phosphorylates NRF2 [85] although the inhibitory effects of p38α/β on NRF2 have been reported [357]. Phosphorylation of myocyte-specific enhancer factor 2 (MEF2) and CCAAT-enhancer binding protein (C/EBP)β [354] has also been reported, thereby triggering the expression of a plethora of genes involved in cell type-specific differentiation. However, AP-1-type transcription factors constitute the main targets for these MAPKs, and a more elaborate description of the target genes of these transcription factors is provided below.

##### *Proliferation*

JUN and ATF2 stimulate cell proliferation by upregulating the production of cyclins D, E, and A; EGFR; heparin-binding EGF (HBEGF); and keratinocyte growth factor (KGF) [358–361]. Additionally, cell cycle inhibitors p53, p19^ARF^, and p21^CIP1^ are downregulated by JUN [362–365]. Contrastingly, JUN and ATF2 also upregulate several cell cycle inhibitors such as the retinoblastoma 1 protein (RB1), GADD 45α and β, and p16^INK4A^ [366, 367].

##### *Angiogenesis and invasion*

Stimulation of angiogenesis and invasion are the result of proinflammatory cytokines, growth factors, and extracellular matrix modifiers that ultimately stimulate tumor regrowth. ATF2 and FOS appear to play major roles in the production of proteins involved in these pathways. Moreover, *c*-*FOS* is subject to upregulation by CREB, a member of the ATF2-like subfamily, which amplifies the FOS pathway [368]. FOS stimulates inflammation by facilitating the production of VEGF, urokinase-type plasminogen activator (uPA), uPA receptor (uPAR), MMP1, MMP3, methionyl-tRNA synthetase 1 (MTS1), Kelch-related protein 1 (KRP1), ferritin repressor protein (FRP), ezrin (EZR), and tropomyosin (TPM) 3 and 5b (reviewed in [262]). ATF2 further contributes to the generation of a proinflammatory state by mediating the production of platelet derived growth factor receptor A (PDGFRA) [369], MMP2 [370], TNF-α [371], IFN-γ [372], and HSP90A5 [373]. In addition, CREB also induces many cytokines such as IL-2, IL-6, IL-10, and TNF-α to cause inflammation that in turn stimulates angiogenesis and invasion [374]. Besides directly stimulating apoptosis, many of the abovementioned cytokines are involved in stimulating immune cells to release a multitude of angiogenic factors *via* NF-κB (Section 3.2) and AP-1 transcription factors (Section 3.4).

##### Apoptosis

In addition to stimulating inflammation and proliferation, AP-1 transcription factors also regulate apoptosis following an oxidative insult. JUN regulates the transcription of antiapoptotic BCL2 family members BCL2, BCL3, BCL-XL, and the proapoptotic BIM [262], the eventual result depending on the extent of damage and the cross-talk between various pathways. Additionally, both JUN and FOS stimulate the extracellular apoptosis pathway by upregulating FAS ligand and FAS receptor (FASR) [262, 375], whereas ATF2 induces the production of TNF-related apoptosis-inducing ligand (TRAIL) [371]. Given the variety of different genes and processes influenced by the AP-1 transcription factor family and the overlap of genes that different family members can induce, the exact effects of AP-1 on overall tumor cell survival or cell death induced by PDT remain difficult to predict. This is because although AP-1 may stimulate tumor growth and survival by mediating cell cycle progression, inflammation, angiogenesis, and migration, AP-1 may also be instrumental in the induction of apoptosis *via* the upregulation of FAS, FASL, and TRAIL, as well the differential regulation of BCL2 protein family members.

##### *Additional effects of p38*^***MAPK***^

To assist in transcription, p38^MAPK^ activates mitogen- and stress-activated protein kinases (MSK) 1 and 2 that phosphorylate histone H3 to enhance chromatin remodeling and transcription factor binding to DNA [376]. The activation of MAPK interacting kinases (MNK) 1 and 2 by p38^MAPK^ further facilitates mRNA translation by phosphorylating the eukaryotic translation initiation factor (EIF)4E that binds RNA and targets it to ribosomes [377], whereas MSK1 contributes to mRNA translation by inactivating the EIF4E inhibitor 4E-binding protein 1 (4EBP1) [378]. Other functions of MSK1/2 include the phosphorylation and activation of transcription factors ATF1, CREB [379], as well as several other transcription factors (*e.g*., NF-κB, ETS variant 1, and high mobility group nucleosome binding domain 1). Through these transcription factors, MSKs upregulate the transcription of *JUN* and *FOS* [379] and contribute to inflammation and survival by upregulating *IL*-*6* and *RELA* (see NF-κB, Section 3.2) [376].

p38α/β activity appears to stimulate cell motility by phosphorylation of MAPK-activated protein kinases 2 and 5 (MK2, MK5) [380]. When activated by p38^MAPK^, these kinases phosphorylate HSP27, causing HSP27 dimerization and consequent binding to the actin cytoskeleton—a phenomenon associated with heightened cell motility in human umbilical vein endothelial cells [381]. Thus, this activity of p38α/β may stimulate tumor cell survival by promoting angiogenesis, invasion, and metastasis.

p38α/β can have positive and negative effects on the cell cycle through the activation of MK2 and 5. MK2 halts the cell cycle by phosphorylating and activating the cell division cycle (CDC) proteins CDC25B and CDC25C, which can functionalize the G2/M checkpoint and arrest the cell cycle in the presence of DNA damage. MK5 promotes senescence by phosphorylating p53 and inhibiting the expression of c-MYC, but also stimulates proliferation by sequestering ERK3 in the cytoplasm [382]. MK2 negatively regulates p53 by phosphorylating the p53 ubiquitin ligase MDM2 (mouse double minute 2, human homologue) and inhibits CDCs to stimulate proliferation despite DNA damage (reviewed in [383]). Other, but less studied effects of p38^MAPK^ include the upregulation of HIF-1α [384] and COX-2 [385] (Sections 3.2 and 3.3), suggesting a survival-promoting role for p38^MAPK^. This has been corroborated in a recent study by Rubio *et al*., in which p38^MAPK^ was implicated in the removal of ubiquitin aggregates *via* autophagy and activation of NRF2 after hypericin-PDT that led to increased survival of fibroblasts [386].

##### Prolonged downstream effects of ASK1 activation

Prolonged activation of JNK stimulates apoptosis. Prolonged JNK1 activation is a signal for extensive cell damage that triggers apoptosis *via* TNF-α and degradation of the caspase 8 inhibitor CFLAR [387, 388]. Apoptosis is further promoted through the inhibition of antiapoptotic BCL2 protein family members BCL2, BCL-XL, and MCL-1 [389, 390] in combination with activation of proapoptotic BAX, BAK, BIM, BCL2-modifying factor (BMF), and BID (yielding JNK-cleaved BID or jBID) [391–393]. In addition, JNK1 stabilizes the tumor suppressor protein p53 to stimulate apoptosis and cell cycle arrest in response to DNA damage [345, 394]. Prolonged activation of JNK1 and consequent cell death signaling is induced by prolonged oxidative stress, depleted antioxidants and impaired survival responses (*e.g*., reduced activity of NRF2 and NF-κB), or TNF-α signaling combined with oxidative stress (Fig. 8). Similarly, in response to phorbol 12-myristate 13-acetate and ionomycin, transient activation of JNK1 was associated with survival of human Jurkat T-cells, whereas prolonged activation of JNK1 (phorbol 12-myristate 13-acetate, ionomycin, and UV-C irradiation) induced cell death [395]. In primary rat mesangial cells, TNF-α treatment alone induced transient JNK1 activation that did not result in loss of cell viability. Conversely, a combined treatment of TNF-α with either actinomycin D or cycloheximide resulted in prolonged JNK1 activation and major decreases in cell viability [396]. With the use of mouse embryonic fibroblasts (MEFs) derived from Traf2^−/−^ and Traf6^−/−^ mice, Noguchi *et al*. revealed that Traf2 and Traf6 (typically activated *via* TNFR) were essential for the induction of H_2_O_2_-induced cell death [340], thereby indicating that simultaneous exposure of cells to TNF-α/TNFR signaling and oxidative stress may facilitate prolonged ASK1 signaling with sustained activation of JNK1. ROS were an essential second messenger for TNF-α-induced apoptosis in murine L929 cells, as the induction of apoptosis in murine L929 cells following combined H_2_O_2_ and TNF-α treatment could be completely prevented by the antioxidant N-acetylcysteine [341]. TNF-α inhibited ASK1/TRX interaction [341]—most likely *via* binding of TRAFs [340]—which could be reversed upon the addition of N-acetylcysteine, suggesting an essential role for oxidants in the induction of TNF-α-mediated apoptosis [341]. Downstream of TNF-α, TRAF2 and RIP1 induced apoptosis in MEFs *via* JNK1 activation, although the involvement of ASK1 itself was not investigated [397]. The induction of cell death *via* TNF-α was prevented when NF-κB was activated in MEFs [350]. NF-κB decreased TNF-α-induced ROS formation, thereby preventing prolonged JNK1 activity and consequent cell death in MEFs [189]. A similar effect was found using PDT, in which inhibition of NF-κB with Bay 11-7082 in human glioblastoma cells augmented TNF-α-induced tumor cell death following PDT [273]. However, the induction of necrosis by ALA-PDT of human glioblastoma cells occurred *via* RIP1 and RIP3 in an NF-κB-independent manner [64].

##### Induction of transient JNK1 activity prevents apoptosis

Several downstream genes regulated by NRF2 and NF-κB in turn affect the transient activity of JNK1 by inhibiting its function to facilitate cell survival (Fig. 8). Since these gene products need to be transcribed and translated before being able to downmodulate JNK1, there is a time period during which JNK1 is active. GSTp is produced downstream of the NRF2 pathway and inhibits JNK1 to stimulate cell survival [398–402] or else transiently inactivates p38^MAPK^ to mediate survival of oxidatively stressed murine 3T3 fibroblasts [402]. Moreover, the NF-κB and AP-1 target genes GADD45α and β limit the activity of JNK1 by binding and inhibiting MKK7 and 4 [403, 404]. Conversely, GADD45α and β increase the activity of p38^MAPK^, whereby the combined effects of JNK1 inhibition and p38 activation protected hematopoietic cells from UV-induced apoptosis [403]. Upregulation of A20 and XIAP by NF-κB leads to blocked JNK1 activity *via* an unknown mechanism (reviewed in [405]).

#### Role of the ASK1 pathway in PDT

Direct ASK1 activation following PDT has never been demonstrated, so the involvement of this pathway in response to PDT can only be deduced from the effects on downstream kinases and transcription factors. Considerable increases in *c*-*FOS* and *c*-*JUN* mRNA levels were found after porfimer sodium-PDT of RIF-1 cells. Levels of mRNA peaked 90 min post-PDT, after which mRNA levels gradually dropped to baseline during the subsequent 8 h [406]. Furthermore, protein kinase inhibitors such as staurosporine effectively blocked the synthesis of *c*-*FOS* mRNA, hinting toward the involvement of upstream kinases p38^MAPK^ and JNK [406]. Activation of JNK and p38^MAPK^, but not other MAPKs such as ERK1 or ERK2, in benzoporphyrin derivative-PDT-subjected murine PAM212 keratinocytes confirmed the involvement of the AP-1 response. The activation of JNK and p38^MAPK^ was abrogated in the presence of antioxidants [407]. Activation of the AP-1 response in the PAM212 keratinocytes was further confirmed by ATF2 and JUN phosphorylation following PDT [407].

#### Inhibition strategies of ASK1 and its downstream targets

Since the discovery of their involvement in the response to PDT, the effects of the MAPKs and AP-1 proteins on the biological fate of PDT-affected cancer cells have been elusive or inconsistent. Two types of approaches can be used to inhibit the ASK1 pathway. The first approach is to inhibit the function of AP-1 transcription factors, while the other method prevents AP-1 activation through inhibition of the upstream kinases JNK and p38^MAPK^. Direct chemical inhibition of AP-1 transcription factors is possible with retinoic acid and its analog SR11302 (Table 1). More specifically, retinoic acid or retinoic acid analogs are able to inhibit ATF2 [408] and c-Jun [409]. It was shown that these inhibitors were efficient in blocking AP-1 activity, which prevented tetradecanoyl phorbol acetate-induced papilloma formation in mice [152, 410]. This illustrates the potential for a treatment modality in which retinoic acid is used to sensitize tumor cells to PDT. However, to date no studies have employed direct inhibition of AP-1 in conjunction with PDT.

Indirect inhibition of AP-1 activation has been achieved with inhibitors of JNK and p38α/β. JNK1 can be inhibited by the anthrapyrazole SP600125 (Table 1), even though this compound also mildly impairs the function of p38β and ERK2 [153]. The pyridinyl imidazole derivatives SB202190, SB203580, and PD169316 have been used for the selective inhibition of p38α/β, but not the δ or γ isozymes (Table 1). The JNK and p38^MAPK^ inhibitors all compete with ATP for the ATP-binding domain of the kinases, resulting in the reversible inhibition of kinase activity [154]. However, the roles of these kinases are rather unpredictable and the effects of inactivation are dependent on the cell type and the extent of damage. The contradictory effects that these MAPKs can have on tumor cell survival following PDT are shown in Table 2. The data reveal that p38^MAPK^ and JNK induce both survival and apoptosis but also suggest that the inhibition of p38α/β augments tumor cell death, whereas inhibition of JNK either has no effect or impairs PDT-induced cell death. This is in line with the dichotomous effects of JNK1 activation and the cytoprotective function of p38α/β as described in Section 3.4.2.2 Prolonged downstream effects of ASK1 activation, in which p38α/β mainly functions as activator of AP-1 and facilitators of transcription. Therefore, it can be deduced that JUN and ATF2 (both downstream of JNK) do not play a significant role in cell survival, but FOS (only activated by p38^MAPK^) may in fact be a decisive factor for cell survival. Alternatively, in a recent study by Rubio *et al*., p38^MAPK^ was implicated in the removal of ubiquitin aggregates *via* autophagy and activation of NRF2 after hypericin-PDT, which resulted in increased survival of fibroblasts [386].

**Table 2:** Studies on human cell lines in which p38^MAPK^ and/or JNK have been chemically inhibited during PDT are summarized

Inhibitor	Dose (μM)	Cell type	Photosensitizer	Fluence	Light source	Outcome	Reference
PD169316	25	HELA ovarian carcinoma	Hypericin (0.125 μM)	4 J/cm^2^	Not specified	*Inhibition of p38 induced apoptosis in JNK-inhibited cells*	[411]
SEK-AL/TAM67 transfection						*Inhibition of JNK induced apoptosis*	
SB202190	15	LYR mouse lymphoma	Pc4 (0.5 μM)	3 J/cm^2^	>600 nm	**Inhibition of p38**α **and β2 prevented apoptosis**	[412]
SB202190	15	CHO Chinese hamster ovary	Pc4 (0.5 μM)	10 J/cm^2^	>600 nm	**Inhibition of p38**α **and β2 prevented apoptosis**	
PD169316	1	T24 bladder cancer	Hypericin (0.15 μM)	4 J/cm^2^	530–620 nm	*Inhibition of p38 induced apoptosis*	[244]
Gene knockout		GM38A fibroblast	Photofrin (10 μg/mL)	0.27 J/cm^2^	630 nm	Knockout of p38 had no effect	[413]
Gene knockout		LFS087 Li-Froumeni syndrome	Photofrin (7.5 μg/mL)	0.27 J/cm^2^	630 nm	Knockout of JNK had no effect	
PD169316	1	T24 cocultured human umbilical vein endothelial cells	Hypericin (0.15 μM)	4 J/cm^2^	530–620 nm	*Inhibition of p38 prevented T24-cocultured HUVEC migration*	[243]
PD169316	0.1	MEF mouse embryonic fibroblasts	Hypericin (0.5 μM)	2.7 J/cm^2^	530–620 nm	*Inhibition of p38 induced apoptosis*	
PD169316	1	T24 bladder cancer	Hypericin (0.15 μM)	4 J/cm^2^	530–620 nm	*Inhibition of p38 induced apoptosis*	[85]
SP600125	1					Not reported	
PD169316	1	T24 bladder cancer	Hypericin (0.15 μM)	4 J/cm^2^	530–620 nm	*Inhibition of p38 induced apoptosis*	[414]
SB202190/SB203580	1 / 12	RIF1 murine radiation- induced fibrosarcoma	Photofrin (25 μg/mL)	315 J/cm^2^	Not specified	*Inhibition of p38α and β2 induced apoptosis*	[202]
SP600125	20					Inhibition of JNK had no effect	
SB203580	20	NuTu-19 rat epithelial ovarian cancer	TPPS2A (2 μg/mL)	1.35 J/cm^2^	435 nm	**Inhibition of p38**α **and β2 prevented apoptosis**	[415]
SP600125	5					*Inhibition of JNK induced apoptosis*	
SB202190	10	HK-1 nasopharyngeal squamous cell carcinoma	Hypericin (1 μM)	0.2 J/cm^2^	590 nm	*Inhibition of p38α and β2 induced apoptosis*	[416]
SB203580	10					*Inhibition of p38α and β2 induced apoptosis*	
SP600125	10					Inhibition of JNK had no effect	
SP600125	0.5	HepG2 hepatocellular carcinoma	Pheophorbide a (0.75 μM)	84 J/cm^2^	610 nm	**Inhibition of JNK prevented apoptosis**	[417]
PD169316	2.5	CNE-2 nasopharyngeal carcinoma	Zn-BC-AM (1 μM)	1 J/cm^2^	682 nm	*Inhibition of p38 induced apoptosis*	[418]
SB203580	10					**Inhibition of p38α and β2 prevented apoptosis**	
SP600125	5					Inhibition of JNK had no effect	
SB203580	10	Ca9-22 oral cancer	ALA (1 mM)	4 J/cm^2^	635 nm	*Inhibition of p38α and β2 induced apoptosis*	[275]
SP600125	10					**Inhibition of JNK prevented apoptosis**	
PD169316	5	T24 bladder cancer	Hypericin (0.15 μM)	1.6 J/cm^2^	White light	*Inhibition of p38 induced apoptosis*	[386]
Gene knockout		MEF mouse embryonic fibroblasts	Hypericin (0.125 μM)	0.4–0.8 J	White light	*Knockout of p38α induced apoptosis*	
SB203580	10	MCF7 human mammary adenocarcinoma	Photofrin (5 μg/mL)	0.36 J/cm^2^	635 nm	*Inhibition of p38α and β2 prevented COX-2 accumulation*	[170]

The outcome represents the prosurvival (italics) or antisurvival (bold) role of the MAPK on tumor cells following PDT. With respect to the PDT strategy, the minimally effective light dose or the maximal dose at which no effect was observed is given

#### Concluding remarks

The ASK1 pathway is one of the most complicated pathways activated by PDT. Although ASK1 itself has, to our knowledge, never been investigated in the context of PDT, many of its downstream targets have often been implicated in cellular responses to PDT. However, the effects of this pathway are highly divergent, ranging from stimulation of survival to causing inflammation and stimulation of cell death. It is arguable that JNK1 has a particularly important role in the stimulation of apoptosis in cells exposed to severe and prolonged oxidative stress or concomitant TNF-α signaling. Experimental evidence regarding the inhibition of JNK-1 and p38α/β is in agreement with this hypothesis. Survival signaling as a result of transient JNK and p38^MAPK^ activity may arise from the phosphorylation of AP-1 transcription factors and the triggering of the immediate early survival response. Therefore, given the dichotomous nature of the ASK1-MAPK pathway, it is hypothesized that pharmacological inhibition of AP-1 transcription factor activation could improve PDT efficacy, while the function of the MAPKs JNK and p38^MAPK^ should remain intact.

### The proteotoxic stress response

ROS production by PDT primarily results in the oxidation of lipids and proteins, leading to protein misfolding or formation of protein aggregates [27]. These forms of proteotoxic stress trigger transcriptional responses that are collectively known as the UPR, which is considered a form of ER stress. The UPR is mediated by several transcription factors that include inositol-requiring protein 1 (IRE1), ATF6, and protein kinase RNA-like ER kinase (PERK). Although generally not included as a part of the UPR, unfolded proteins generated as a result of oxidative and proteotoxic stress also activate HSF transcription factors, which aid in alleviating ER stress [419]. The HSF transcription factors facilitate an adaptive response that enables protein refolding and degradation of protein aggregates *via* the upregulation of chaperones and inhibition of protein neogenesis (reviewed in [420]).

It can be seen that, when the adaptive responses to unfolded proteins and ER stress are constitutively active in tumor cells, the threshold for the ROS-mediated induction of cell death will be higher. HSFs trigger the production of HSPs to assist in protein refolding, protein complex formation, or protein degradation to alleviate the proteotoxic stress [421, 422]. Clinical evidence has shown that HSF1 and its downstream products HSP27, 70, and 90 are often constitutively activated in tumors [423]. The same constitutive activation was found for components of the IRE1 and PERK pathways [424], thus pointing toward a predisposition of tumor cells to be able to cope with PDT-induced proteotoxic stress.

#### Activation mechanisms of the proteotoxic stress response

**Fig. 9:**
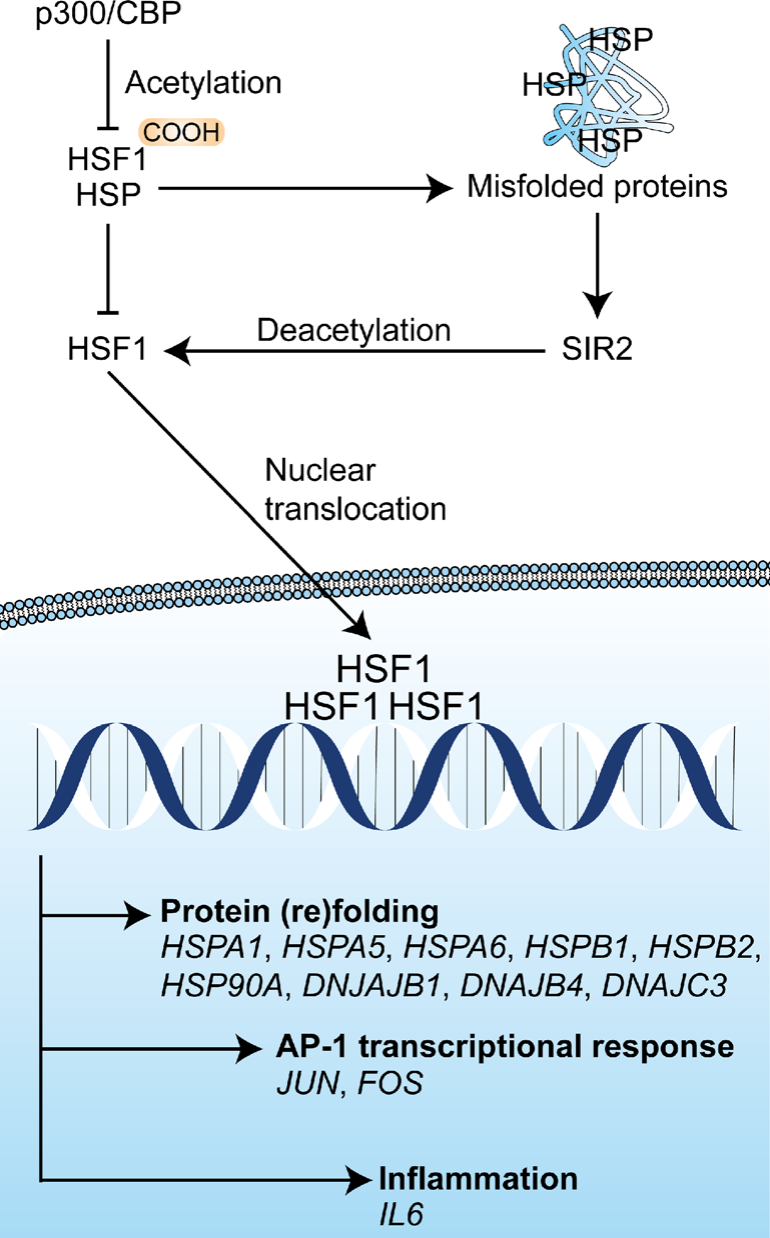
Mechanism of HSF1 activation. Monomeric HSF1 is kept inactive in the cytosol *via* acetylation by p300/CREB binding protein. The cytosolic monomers interact with HSP40, 70, or 90, which prevents DNA binding and transactivation. Proteotoxic stress titrates HSPs away from HSF1 and stress-induced SIR2 deacetylates the HSF1 monomers. Subsequently, HSF1 forms homotrimers, translocates to the nucleus, and binds genomic heat shock elements to facilitate target gene expression. The target gene products are mainly involved in protein (re)folding, but also enforce the immediate early stress response and inflammation

**Fig. 10:**
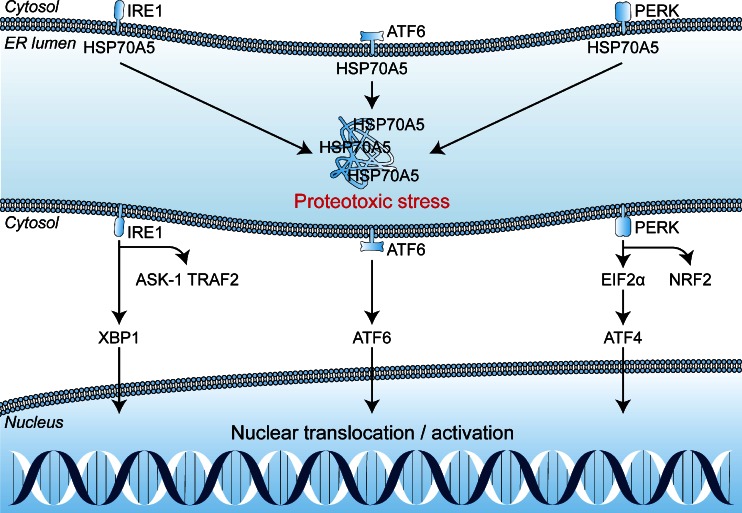
Activation mechanism of the ER stress response as a result of proteotoxic stress. Unfolded protein detectors IRE1, PERK, and ATF6 are sequestered by HSP70A5. Upon proteotoxic stress due to protein oxidation, misfolding, or formation of protein aggregates, HSP70A5 is recruited toward the misfolded proteins. The ER stress detectors are subsequently activated, leading to the initiation of XBP1, ATF4, and ATF6 transcription factor function

Of the four HSFs isoforms 1–4, HSF1 is the most important [421] and will be discussed in the context of the proteotoxic stress response. Under nonstressed conditions, HSF1 forms inactive monomers in the cytoplasm that are acetylated by p300/CREB binding protein (CBP). HSP40, 70, and 90 form complexes with HSF1 and negatively regulate either its transactivation mechanism (HSP90) or its DNA-binding capacity (HSP40 and HSP70) [421, 425]. Whenever HSPs need to be engaged to combat proteotoxic stress by ROS or hypoxia, HSPs dissociate from the HSF complex and bind to misfolded proteins. This binding relieves the negative regulation of HSF, leading to HSF homotrimerization and activation [421]. Moreover, stress-induced sirtuin (SIR2) deacetylates HSF and maintains the complex in a state capable of binding DNA [425] (Fig. 9). Additionally, JNK2 has been implicated in phosphorylating the transactivation domain of HSF1, contributing to its activation and subsequent binding to genomic heat shock elements [426]. Other sensors of the ER stress response include IRE1, ATF6, and PERK. These proteins are embedded in ER membranes and are activated when their chaperones (mainly HSP70A5, also known as glucose-regulated protein 78 (GRP78) and binding immunoglobulin protein (BIP)) are recruited away to bind to unfolded proteins (Fig. 10).

#### Downstream effects of HSF1, IRE1, PERK, and ATF6

##### HSF1

HSF1 induces the expression of a plethora of heat shock proteins (including but not limited to HSP27, HSP40 (DNAJ), HSP70, and HSP90 [427]), which are molecular chaperones that assist in the folding, translocation, and complexation of newly translated proteins (Fig. 9). Increased expression of HSPs enhances the survival of cells that have been stressed by prooxidative conditions, hypoxia, or chemotherapeutic agents [428]. Consequently, the constitutive activation of the heat shock response in tumors (as a result of constitutive mild stress) is frequently observed and protects tumor cells from the cytotoxicity involved in different kinds of cancer therapy (chemotherapy, radiotherapy, and PDT) [429, 430]. HSF1 also induces *JUN* and *FOS* (Fig. 9), thereby reinforcing the AP-1-induced immediate early stress response (Section 3.4.2.2 Prolonged downstream effects of ASK1 activation) [431, 432]. Other, less studied effects of HSF1 are the induction of *IL6* (Fig. 9) and the consequent transcriptional repression of *TNFA*, *IL1B*, *NOS2*, *CCL5*, *IL8*, *C5*, and *ICAM1* by inhibiting NF-κB activation (reviewed in [433]). The HSF1 pathway most likely raises the level of intracellular stress necessary for IKK activation and IκBα degradation that are required to activate NF-κB [433]. The reduction of proteotoxic stress emanating from oxidatively modified proteins has been attributed to the chaperone function of HSPs, which tend to prevent protein denaturation and aggregation, assist in the correct refolding of affected proteins, promote GSH reduction, and maintain proteasome function (reviewed in [428, 429, 434]). Moreover, HSP70 prevents apoptosis by reducing JNK1 phosphorylation and BID-mediated mitochondrial membrane permeabilization; preventing caspase 8 activation by FADD; lessening the mitochondrial localization of BAX; inhibiting cleavage of procaspases 3, 7, and 9; and binding apoptosis-inducing factor (AIF) and a variety of other apoptotic effectors [429]. HSP27 is also an inhibitor of apoptosis by sequestering cytochrome c when released from the mitochondria, preventing the mitochondrial release of second mitochondria-derived activator of caspases (SMAC), blocking the activation of the proliferative PI3K-AKT pathway that enables cells to divert energy consumption toward relieving the ER stress, and stimulating prosurvival NF-κB signaling [430]. The latter effect is somewhat controversial given the consensus that downregulation of many NF-κB target genes can be attributed to the HSP-dependent inhibition of NF-κB activation, suggesting a complicated relationship between the HSF pathway and the NF-κB pathway.

##### IRE1, ATF6, and PERK

**Fig. 11:**
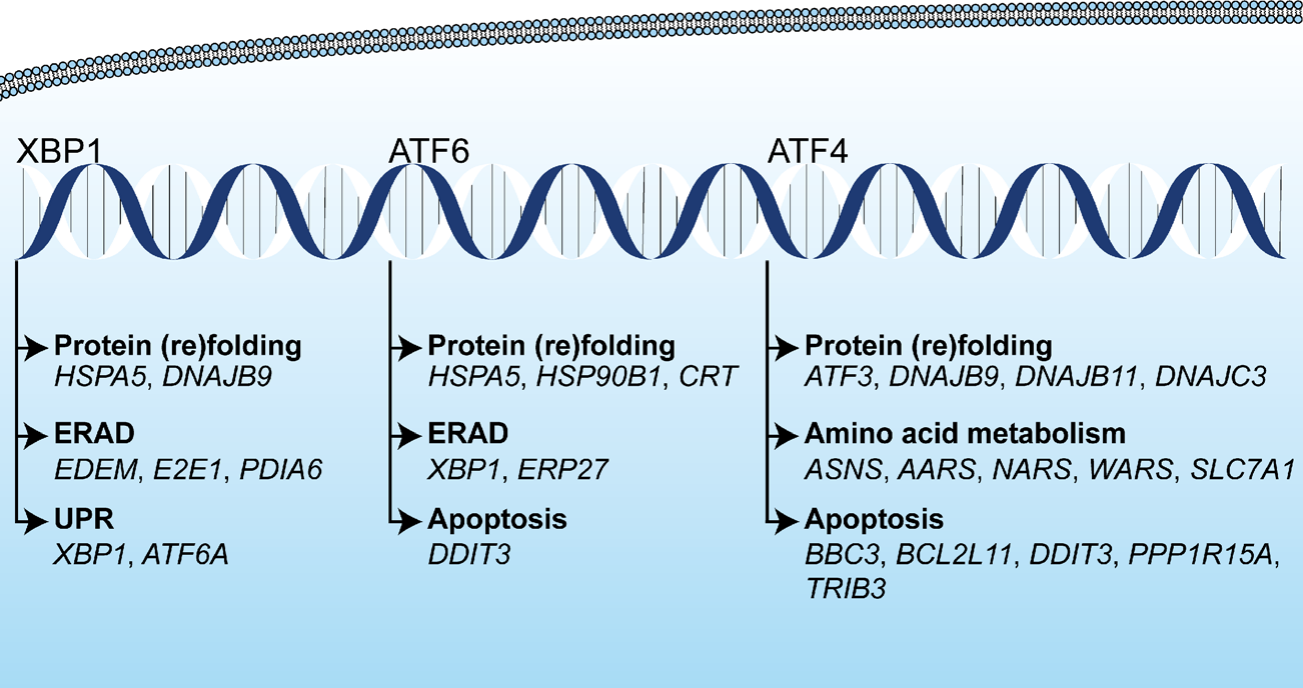
Transcriptional regulation of genes induced by XBP1, ATF6, and ATF4 in response to proteotoxic stress. XBP1 stimulates protein (re)folding, ERAD, and amplifies the UPR. ATF6 also promotes protein (re)folding and ERAD, but also stimulates apoptosis by upregulating *DDIT3* (CHOP). ATF4 ameliorates proteotoxic stress by upregulating *ATF3* and a plethora of *DNAJ* genes (encoding various isoforms of HSP40). ATF4 additionally upregulates genes involved in amino acid metabolism that include, but are not limited to, asparagine synthetase (*ASNS*), alanyl-tRNA synthetase (*AARS*), asparagyl-tRNA synthetase (*NARS*), tryptophanyl-tRNA synthetase (*WARS*), and the cationic amino acid transporter *SLC7A1*. ATF4 additionally upregulates proapoptotic genes *BBC3*, *BCL2L11*, *DDIT3*, *PPP1R15A*, and *TRIB3*

Besides HSF1, downstream events related to proteotoxic stress are also induced by the IRE1, ATF6, and PERK. IRE1 has kinase activity and RNAse activity *via* which it stimulates autophagy and apoptosis. The cytosolic domain of IRE1 complexes with TRAF2 to activate ASK1, resulting in prolonged, proapoptotic JNK1 activation. Autophagy is stimulated by IRE1 *via* the splicing of *XBP1* mRNA, resulting in the accumulation of an active XBP1 transcription factor. XBP1 upregulates the production of HSP70A5, protein disulfide-isomerase (PDI)P5, HSP40B9, ubiquitin-conjugating enzyme E2E1, and the ER degradation-enhancing α-mannosidase-like protein 1 (*EDEM1*) [435] (Fig. 11) that all aid in refolding and degradation of misfolded proteins, a process termed ER-associated degradation (ERAD) [436]. ERAD is a form of autophagy through which terminally misfolded proteins and protein complexes are targeted for proteasomal degradation, eventually reducing proteotoxic (ER) stress [436]. Additional target genes of XBP1 include *XBP1* and *ATF6A* as well as several other genes with a diverse range of functions [435] (Fig. 11).

ATF6 is also activated by proteotoxic stress and initiates the transcription of chaperones and ERAD-associated genes. These chaperone genes include *HSPA5* (HSP70A5), *HSP90B1*, and *CRT* (calreticulin, CRT). ATF6 additionally triggers the expression of ERAD-stimulating genes such as *XBP1*, *PDI*, yeast Der1-like protein (*DERL1*), homocysteine-induced ER-protein (*HERP*), synovial apoptosis inhibitor 1 (*SYVN1*), and suppressor of Lin-12-like (*SEL1L*) [437]. ATF6 also upregulates C/EBP homologous protein (CHOP, encoded by *DDIT3*) to promote apoptosis [436, 438] (Fig. 11).

Activated PERK phosphorylates and activates NRF2 (Section 3.1) and EIF2α, resulting in activation of the antioxidant stress response and general inhibition of translation yet the selective translation of ATF4 mRNA. In turn, ATF4 stimulates both apoptosis and survival. It upregulates the expression of proapoptotic proteins such as CHOP, p53-upregulated modulator of apoptosis (PUMA, or BCL2-binding component 3 (*BBC3*)), GADD34 (or protein phosphatase 1, regulatory subunit 15a (*PPP1R15A*), tribbles-related protein 3 (*TRIB3*), and BIM (*BCL2L11*) [437]. Survival is promoted *via* stimulation of amino acid metabolism, protein (re)folding, and restoration of redox homeostasis [439, 440] (Fig. 11). The latter function is achieved *via* HO-1 upregulation by complex formation with NRF2 [441]. Interestingly, ATF4 is activated by hypoxia and plays an important role in resistance to cancer therapy in a similar fashion to HIF-1 [440]. Interested readers are referred to more elaborate reviews on ER stress and the UPR [420, 425].

#### Role of the proteotoxic stress response in PDT

PDT was found to activate HSF [442, 443] and stimulate the production of HSP70, HSP47, HSP60, and HSP27 [442, 444–450]. Furthermore, high levels of HSP27, HSP60, HSP70, and HSP90 were linked to reduced susceptibility of tumor cells to PDT *in vitro* and *in vivo* [250, 444, 448, 450, 451]. The cytoprotective properties of HSPs after PDT likely arise from the alleviation of proteotoxic stress that ensues protein oxidation. The induction of ER stress by PDT was studied by Szokalska *et al*., who showed that porfimer sodium-PDT leads to extensive protein carbonylation, polyubiquitination, and widening of the ER lumen [27]. Moreover, PDT induced XBP1 activation and upregulation of HSP70A5 and calnexin, whereby ERAD was crucial for the survival of EMT-6 and HeLa cells after PDT *in vitro* and *in vivo* [27]. A microarray looking at the transcriptional response of T24 bladder cancer cells to hypericin-PDT showed significant mRNA upregulation of HSP70A5, 40, 47, 60, 90, 110, CHOP, GADD34, XBP1, PERK, ATF3, and ATF4, providing compelling evidence for the involvement of HSF1 and the UPR in response to PDT [124]. A follow-up study by the same group suggested that PERK-upregulated NOXA was the main instigator of tumor cell death after hypericin-PDT-induced ER stress [452].

With respect to the antitumor immune response, HSP70 in particular has been implicated in immune cell modulation after PDT [199, 422]. Apoptotic cells expressed HSP70 on their plasma membrane after PDT [445], most likely in an attempt to stabilize the plasma membrane or chaperone integral membrane proteins [422]. Moreover, HSP70 can bind protein fragments derived from tumor-specific antigens such as mutated, truncated, or misfolded proteins. When expressed on the plasma membrane or released from necrotic cells, these HSP70/tumor antigen complexes can be taken up by dendritic cells to induce their maturation, activation, and migration to lymph nodes, where they can initiate cross-presentation to naive T-cells and stimulate the formation of tumor-specific CD8^+^ cytotoxic T cells (reviewed in [199]). A similar mechanism in favor of dendritic cell activation has been ascribed to CRT (a downstream gene product of ATF6), which aids in ER protein folding. When CRT is expressed on the outer leaflet of the plasma membrane (ecto-CRT), it is associated with the induction of immunogenic cell death that stimulates antigen presentation and the activation of immune cells. Following hypericin-PDT (hypericin localizes to the ER), T24 cells expressed ecto-CRT in a PERK-dependent manner. Moreover, PERK was essential for the immunogenicity of CT26 cells treated with hypericin-PDT *in vivo* [453]. Thus, the proteotoxic stress response seems to play a key role in mediating an antitumor immune response.

#### Inhibition strategies for the proteotoxic stress response and its downstream targets

It may be postulated that inhibition of HSPs would be beneficial when beneficial for PDT outcome given the role of HSPs in tumor cell survival [422]. However, their role in promoting the antitumor immune response also suggests that HSP inhibition may be detrimental [199]. Ferrario and Gomer used the geldanamycin derivative 17-AAG [155] to inhibit the function of HSP90 (Table 1) during PDT and found a reduction in protein levels of survivin, VEGF, phospho-AKT, and BCL2. A higher cure rate and long-term survival were observed in BA mammary tumor-bearing mice treated with PDT combined with 17-AAG [250, 252]. HSP70 inhibition with the bacterial cytotoxin SubA fused to EGF [160], (Table 1) was recently shown to augment the efficacy of porfimer sodium-PDT in human SW-900 lung cancer cells and DU-145 prostate cancer cells as a result of increased ER stress [454]. Taken together, these results point toward the beneficial effect of HSP inhibition in the enhancement of PDT efficacy. Besides 17-AAG, other HSP90 inhibitors are available and include different geldanamycin derivatives, although these may be associated with liver toxicity [455], as well as the synthetic small molecules CNF-2024/BIIB-021, NVP-AUY922, SN-X5422, and STA-9090 (Table 1), which are undergoing clinical trials [156–159, 456]. However, inhibition of HSP90 typically exacerbates proteotoxic stress that induces HSP70 proteins [457] and may therefore alleviate any beneficial effects of these agents in terms of tumor cell death.

Alternatively or in addition to HSP90 inhibition, HSP70 inhibitors are also available. Schlecht *et al*. recently demonstrated the inhibition of HSP70 and HSC70 (a constantly expressed isozyme of HSP70) using VER-155008, a compound that binds the nucleotide binding domain of these proteins and reduces their ATPase activity (Table 1). In RNAi knockdown experiments, it was shown that concomitant inhibition of HSP70 and HSC70 was necessary to induce tumor cell death [161]. A more effective approach to completely abolish the heat shock response is to block HSF1 activity. KRIBB11 (*N*^2^-(1H-indazole-5-yl)-*N*^6^-methyl-3-nitropyridine-2,6-diamine) is an HSF1 inhibitor that blocks the association between HSF1 and positive elongation factor b, which is required for HSF1 transcriptional activity (Table 1). Accordingly, KRIBB11 was very effective in preventing HCT-116 tumor growth in nude mice [458]. Based on these results, inhibitors of the HSF pathway could be used to elucidate the role of this pathway in PDT and may provide promising approaches to improve PDT efficacy.

 During ER stress, cells deal with the accumulation and aggregation of carbonylated proteins by polyubiquitination and proteasomal degradation. Therefore, Szokalska *et al*. investigated whether inhibition of the proteasome could exacerbate ER stress and increase the extent of cell death after PDT. Indeed, porfimer sodium-PDT on EMT-6 and HeLa cells pretreated with 4 ng/mL bortezomib (binds and inhibits the catalytic center of the 26S proteasome [162], Table 1) for 24 h increased the accumulation of carbonylated proteins and disrupted ERAD, leading to an increased sensitivity of cells to PDT [27]. Similar results were obtained for verteporfin-PDT in combination with bortezomib (2 mg/kg) in a PC-3 mouse xenograft model [459]. Thus, these results attest to the utility of pharmacological interventions in proteasome function as a means to augment ER stress and improve the therapeutic efficacy of PDT. Pharmacological inhibition of IRE1 and ATF6 (but not PERK) is possible with 4-phenylbutyric acid analogs (Table 1), although the exact mechanism has not been elucidated [163]. With respect to PERK, inhibition is possible with the synthetic compound GSK2656157 (Table 1), which competes with ATP to bind PERK specifically, and thus inhibits its kinase activity [164]. However, none of these UPR-inhibiting compounds have been investigated in combination with PDT.

#### Concluding remarks

Proteotoxic stress appears to be a primary response to PDT regardless of cell type and PDT strategy. The degree to which this response is triggered depends somewhat on the photosensitizer localization insofar as ER-localizing photosensitizers such as hypericin are more effective in inducing the UPR than photosensitizers that accumulate in other intracellular venues. While the functional outcome of this pathway may be both protective and destructive in tumor cells, the protective effects of the proteotoxic stress response can be pharmacologically blocked to promote tumor cell death. Inhibition of HSP70 and HSP90 was shown to increase the efficacy of PDT, as did inhibition of the proteasome by exacerbating ER stress. The HSF pathway is an essential component of the UPR in response after PDT. Given its reported induction by hypoxia and its constitutive activation in tumor cells [460], the UPR may protect tumors against anticancer therapies [424] such as PDT. Disrupting the cytoprotective effects of the UPR or interfering with the function of chaperones has been shown to enhance proteotoxic stress and stimulate cellular demise after PDT. Thus, the proteotoxic stress pathway is an important and feasible target for pharmacological interventions to enhance the therapeutic efficacy of PDT.

## Concluding remarks

Tumor cells have the intrinsic ability to adapt to potentially harmful situations, such as those induced by chemotherapy, radiotherapy, and PDT. With respect to PDT, the activation of NRF2, NF-κB, HIF1, ASK1, HSF1, IRE1, PERK, and ATF6 and the effects of their downstream protein and gene targets have been reviewed. Together, these transcription factors and kinases facilitate the survival of tumor cells that suffer from a disrupted redox balance, low oxygen availability, apoptotic signaling, and oxidative damage to proteins.

The pathways that have the highest potential for pharmacological inhibition with the aim to improve the therapeutic efficacy of PDT are those from which no proapoptotic stimuli emerge. In that respect, blocking the NRF2, HIF1, and HSF1 pathways holds the highest potential to reduce the extent of tumor cell survival post-PDT. This is reflected by the substantial amount of evidence in which the inhibition of one or more of the downstream protein products (*e.g*., HO-1, COX-2, HSP70) from these pathways has led to increased efficacy of PDT. Unfortunately, the conclusion is not that straightforward regarding the ASK1 pathway. The ASK1 signaling axis mainly promotes survival *via* transient JNK1 and p38^MAPK^ activity and their induction of the AP-1 transcription factors. However, upon prolonged oxidative stress and corollary TNF-α signaling, JNK1 has potent proapoptotic activity. Thus, selective inhibition of p38α/β, but not the complete ASK1 signaling cascade, may be therapeutically beneficial for PDT, as is evidenced by the available literature on this topic (Table 1). The transcriptional events emanating from the activated UPR transcription factors IRE1, ATF6, and PERK are also challenging with respect to designing a pharmacological inhibition strategy. Whereas no proapoptotic signaling appears to arise from IRE1, both ATF6 and PERK promote apoptosis *via* the induction of, *e.g*., CHOP. Moreover, the multitude of potential target genes and effects make it arduous to predict the results of an inhibition strategy in conjunction with PDT. Thus, there is an explicit need for further investigations regarding the importance of these particular pathways in the cellular response to PDT. Inhibition of the NF-κB pathway appears unwise given its strong proinflammatory function and its potential to induce programmed cell death. It is probable that some downstream targets of this pathway are very strong inducers of tumor cell survival (*i.e*., COX-2 and survivin), yet completely abolishing this pathway has not produced convincing evidence that pharmacological inhibition is feasible in combination with PDT. Thus, the ambiguous downstream effects of the AP-1, UPR, and NF-κB pathways illustrate an obvious pitfall in applying a pharmacological inhibition strategy for these signaling cascades, since blocking a particular pathway also diminishes any proapoptotic effects of that pathway. A less obvious risk is the use of a compound that is capable of scavenging ROS that are produced during the photoexcitation of the intratumoral photosensitizers. This reduces the effective amount of PDT-produced ROS required to induce cell death. Therefore, an extensive photochemical characterization of the compound of interest should be performed prior to further experimentation regarding pathway inhibition and PDT efficacy. Finally, when a suitable compound has been selected and has yielded favorable outcomes, a careful investigation of the prolonged antitumor immune response should be conducted. Many of the pathways discussed in this review induce immune-modulating and angiogenic factors that may negatively affect the antitumor immune response, which is required to facilitate effective removal of the tumor.

Many of the key signaling proteins discussed in this review are constitutively active in tumors and may therefore contribute to a natural resistance to PDT. Therefore, tumors that typically respond poorly to PDT such as nasopharyngeal carcinomas, bladder tumors, and extrahepatic cholangiocarcinomas may be rendered substantially more susceptible to PDT when these adaptive pathways are inhibited. Investigations regarding the constitutive activation of these pathways in the abovementioned tumor types are highly valuable in selecting a suitable pharmacological inhibition strategy.

 In conclusion, the promising investigations in which survival pathway inhibitors are used as (neo)adjuvant agents in PDT are of high importance to cancer patients. A higher PDT efficacy will lead to better disease management, lower morbidity, and prolonged patient survival.

## Abbreviations

2-ME: 2-Methoxyestradiol
3-AT: 3-Amino-1,2,4-triazole
4EBP1: 4E-binding protein 1
ABCG2: ATP-binding cassette subfamily G member 2
ABL: Abelson murine leukemia viral oncogene
AK: Actinic keratoses
ALA: Aminolevulinic acid
AP-1: Activator protein 1
APAF1: Apoptotic protease activating factor 1
APO: Angiopoietin
ARE: Antioxidant response element
ARNT: Aryl hydrocarbon receptor nuclear translocator
ASK1: Apoptosis signal-regulating kinase 1
ATF: Activating transcription factor
PKD: Protein kinase D
BAX: BCL2-associated X protein
BAK: BCL2 homologous antagonist killer
BC: Bladder cancers
BCL: B-cell lymphoma protein
BCR: Breakpoint cluster region protein
bHLH: Basic helix-loop-helix
BID: BH3 interacting domain death agonist
BIP: Binding immunoglobulin protein
BIRC: Baculoviral inhibitor of apoptosis repeat-containing
BNIP3: BCL2/adenovirus E1B 19 kDa protein-interacting protein 3
BNIP3L: BNIP3 ligand
BSO: l-Buthionine sulfoximine
bZIP: Basic leucine zipper domain
C/EBP: CCAAT-enhancer binding protein
CBP: CREB binding protein
CCR7: C-C chemokine receptor 7
CCL: Chemokine C-C motif ligand
CD40L: Cluster of differentiation 40 ligand
CDC: Cell division cycle protein
CES1A1: Carboxylesterase 1A1
CFLAR: Caspase 8 and frequently associated with death domain-like apoptosis regulator
CHOP: C/EBP homologous protein
cIAP: Cytosolic inhibitor of apoptosis protein
CO: Carbon monoxide
COX: Cyclooxygenase
CREB: Cyclic AMP response element
CRT: Calreticulin
CXCL: Chemokine C-X-C motif ligand
DCF: 2′-7′-Dichlorofluorescein
DCFH_2_: 2′-7′-Dichlorodihydrofluorescein
DDC: Diethyl-dithiocarbamate
DHMEQ: Dehydroxymethylepoxyquinomicin
ECLC: Early central stage lung cancers
EDN1: Endothelin 1
EGFR: Epidermal growth factor receptor
EH-1: Microscomal epoxide hydroxylase
EIF: Eukaryotic translation initiation factor
ELK-1: ETS domain containing protein
EM: Esophageal malignancies
EPO: Erythropoietin
ERAD: ER-associated degradation
ERK: Extracellular signal regulated kinase
EZR: Ezrin
FADD: FAS-associated with death domain
FASR: FAS receptor
FGF: Fibroblast growth factor
FIH: Factor inhibiting HIF-1
FOS: FBJ murine osteosarcoma viral oncogene homologue
FRA: FOS-related antigen
FRP: Ferritin repressor protein
GADD: Growth arrested and DNA damage induced protein
GCL: Glutamate-cysteine ligase
GLUT: Glucose transporter
GM-CSF: Granulocyte-macrophage colony stimulating factor
gp130: Glycoprotein 130
GRP78: Glucose-regulated protein 78
GST: Glutathione S-transferase
HBEGF: Heparin-binding EGF
HIF-1: Hypoxia-inducible factor 1
HK: Hexokinase
HO-1: Heme oxygenase-1
HRE: Hypoxia responsive element
HSF1: Heat shock factor 1
HSP70: Heat shock protein 70
HSP90: Heat shock protein 90
ICAM: Intracellular adhesion molecule
IFN: Interferon
IGF: Insulin-like growth factor
IL: Interleukin
IL-6R: IL-6 receptor
IκB: Inhibitor of κB
IKK: IκB kinase
IRE1: Inositol-requiring enzyme 1
JNK: c-Jun N-terminal kinase
KEAP1: Kelch-like ECH-associated protein 1
KGF: Keratinocyte growth factor
KRIBB: *N*^2^-(1H-indazole-5-yl)-*N*^6^-methyl-3-nitropyridine-2,6-diamine
KRP1: Kelch-related protein 1
LDHA: Lactate dehydrogenase A
LUBAC: Linear ubiquitin assembly chain
MAF: Musculoaponeurotic fibrosarcoma oncogene homologue
MAPK: Mitogen-activated protein kinase
MCL-1: Myeloid leukemia cell differentiation protein
MCT: Monocarboxylate transporter
MEF2: Myocyte-specific enhancer factor
MK: MAPK-activated protein kinase
MKK: MAPK kinase
MMP: Matrix metalloproteinase
MNK: MAPK interacting kinase
MRP: Multidrug resistance protein
MSK: Mitogen- and stress-activated protein kinase
MTS1: Methionyl-tRNA synthetase 1
NC: Nasopharyngeal carcinoma
NEMO: NF-κB essential modulator
NF-κB: Nuclear factor κB
NIK: NF-κB inducing kinase
NOS2: (Inducible) nitric oxide synthase 2
NQO1: NAD(P)H:quinine oxidoreductase 1
NRF2: Nuclear factor E2-related factor 2
NSAIDs: Nonsteroidal anti-inflammatory drugs
ODD: Oxygen-dependent degradation domain
PAI1: Plasminogen activator inhibitor 1
PERK: Protein kinase RNA-like ER kinase
PDH: Pyruvate dehydrogenase
PDT: Photodynamic therapy
PDGFRA: Platelet-derived growth factor receptor A
PDI: Protein disulfide isomerase
PFKL: Phosphofructokinase L
PG: Prostaglandin
PGK1: Phosphoglycerate kinase 1
PHD: Prolyl hydroxylase domain protein
PI3K: Phosphatidyl inositol 3 kinase
PKC: Protein kinase C
PKM: Pyruvate kinase (muscle)
PPIX: Protoporphyrin IX
PTGS2: Prostaglandin synthase 2
RARα: Retinoic acid receptor α
RAS: Rat sarcoma protein
REL: Reticuloendotheliosis
RIPK: Receptor-interacting serine/threonine protein kinase
ROS: Reactive oxygen species
SC: Skin cancers
Ser: Serine
sgp130: Soluble gp130
SHIP: SH2-containing inositol 5′-phosphatase
sIL-6R: Soluble IL-6R
SIR2: Sirtuin
SMAC: Second mitochondria-derived activator of caspases
SnPP: Tin PPIX
SOD: Superoxide dismutase
SRC: Sarcoma protein
SRXN1: Sulfiredoxin
STAT3: Signal transducer and activator of transcription 3
TAK1: Transforming growth factor β-activated kinase 1
TBA_2_: Thromboxane A_2_
TGF-β: Transforming growth factor β
Thr: Threonine
TNF-α: Tumor necrosis factor α
TNFR: TNF receptor
TPM: Tropomyosin
TRADD: TNFR associated with death domain
TRAF: Tumor necrosis factor receptor associated factor
TRAIL: TNF-related apoptosis-inducing ligand
TRX: Thioredoxin
uPA: Urokinase-type plasminogen activator
uPAR: uPA receptor
UPR: Unfolded protein response
VCAM: Vascular cell adhesion molecule
VEGF: Vascular endothelial growth factor
VHL: Von Hippel-Lindau protein
XBP1: X-box binding protein 1
XIAP: X-linked inhibitor of apoptosis
ZnPP: Zinc PPIX
